# Yolk sac, but not hematopoietic stem cell–derived progenitors, sustain erythropoiesis throughout murine embryonic life

**DOI:** 10.1084/jem.20201729

**Published:** 2021-02-10

**Authors:** Francisca Soares-da-Silva, Laina Freyer, Ramy Elsaid, Odile Burlen-Defranoux, Lorea Iturri, Odile Sismeiro, Perpétua Pinto-do-Ó, Elisa Gomez-Perdiguero, Ana Cumano

**Affiliations:** 1 Lymphocytes and Immunity Unit, Immunology Department, Institut National de la Santé et de la Recherche Médicale U1223, Institut Pasteur, Paris, France; 2 Instituto de Investigação e Inovação em Saúde and Instituto Nacional de Engenharia Biomédica, Universidade do Porto, Porto, Portugal; 3 Instituto de Ciências Biomédicas Abel Salazar, Universidade do Porto, Porto, Portugal; 4 Graduate Program in Areas of Basic and Applied Biology, Instituto de Ciências Biomédicas Abel Salazar, Universidade do Porto, Porto, Portugal; 5 Macrophages and Endothelial Cells, Department of Developmental and Stem Cell Biology, UMR3738 Centre national de la recherche scientifique, Institut Pasteur, Paris, France; 6 Université Paris Diderot, Sorbonne Paris Cité, Cellule Pasteur, Paris, France; 7 Sorbonne Université, Collège Doctoral, Paris, France; 8 Institut Pasteur, Transcriptome and EpiGenome, Biomics Center for Innovation and Technological Research, Paris, France

## Abstract

In the embryo, the first hematopoietic cells derive from the yolk sac and are thought to be rapidly replaced by the progeny of hematopoietic stem cells. We used three lineage-tracing mouse models to show that, contrary to what was previously assumed, hematopoietic stem cells do not contribute significantly to erythrocyte production up until birth. Lineage tracing of yolk sac erythromyeloid progenitors, which generate tissue resident macrophages, identified highly proliferative erythroid progenitors that rapidly differentiate after intra-embryonic injection, persisting as the major contributors to the embryonic erythroid compartment. We show that erythrocyte progenitors of yolk sac origin require 10-fold lower concentrations of erythropoietin than their hematopoietic stem cell–derived counterparts for efficient erythrocyte production. We propose that, in a low erythropoietin environment in the fetal liver, yolk sac–derived erythrocyte progenitors efficiently outcompete hematopoietic stem cell progeny, which fails to generate megakaryocyte and erythrocyte progenitors.

## Teaser

Yolk sac erythropoiesis is not replaced by hematopoietic stem cell–derived progeny up until birth. Yolk sac–derived erythroid progenitors require 10-fold lower concentrations of erythropoietin for differentiation, which confers a competitive advantage over progenitors of hematopoietic stem cell origin.

## Introduction

Erythrocytes are the most abundant cells in circulation. They transport oxygen and have a half-life of around 22 d in mice and 120 d in humans. Therefore, constant production in the bone marrow (BM) is required to maintain the numbers of circulating RBCs.

Erythropoiesis is the process whereby hematopoietic stem cells (HSCs) progressively differentiate into megakaryocyte/erythrocyte progenitors (MEPs) and later into lineage-committed erythroid progenitors, immature burst-forming unit–erythroid cells (BFU-Es), and the more mature CFU-erythroid cells (CFU-Es). CFU-Es successively progress in differentiation through nucleated proerythroblast, basophilic, polychromatophilic, and orthochromatic stages, enucleation, and formation of RBCs. The distinct stages of erythroid differentiation are characterized by changes in surface expression of the progenitor marker Kit, of the transferrin receptor CD71, of the adhesion molecule CD44, and of the mature erythroid marker Ter119 (Kina et al., 2000; Aisen, 2004; Chen et al., 2009).

During mouse embryogenesis, three overlapping hematopoietic waves emerge in distinct anatomical sites. The first blood cells arise in the yolk sac (YS) blood islands at embryonic day (E) 7.5 and belong to the macrophage, erythroid, and megakaryocytic lineages (Palis et al., 1999). Primitive erythrocytes are large nucleated cells that express a specific pattern of embryonic (εy- and βH1-) globins (Kingsley et al., 2006). Erythromyeloid progenitors (EMPs) arise in the YS around E8.5 (Bertrand et al., 2005) and differentiate into erythrocytes, megakaryocytes, macrophages, and other myeloid lineages such as neutrophils, granulocytes, and mast cells, but lack HSC activity (Palis et al., 1999; McGrath et al., 2015a; McGrath et al., 2015b). EMP-derived erythrocytes resemble adult erythrocytes and express embryonic βH1- and adult β1- but no εy-globins (McGrath et al., 2011). HSCs emerge after E8.5 (E8.5–E11.5; Cumano et al., 1996; de Bruijn et al., 2000; Taoudi et al., 2008; Bertrand et al., 2010; Kissa and Herbomel, 2010; Kieusseian et al., 2012) in the major arteries through an endothelial to hematopoietic transition process, rapidly enter circulation, and colonize the fetal liver (FL), where they expand and differentiate, generating the blood lineages. EMPs that arise through a similar process in the YS (Frame et al., 2016; Kasaai et al., 2017) also converge to the FL, where they are identified as Kit^+^CD16/32^+^, in contrast to Kit^+^CD16/32^−^ HSCs.

In *c-Myb* mutants, where primitive hematopoiesis is preserved but HSC-derived hematopoiesis is missing, YS-derived primitive erythrocytes suffice to maintain living embryos up until E15.5 (Mucenski et al., 1991; Tober et al., 2008; Schulz et al., 2012). In the absence of HSC activity, EMP-derived hematopoietic cells maintain viable embryos throughout development up until birth (Chen et al., 2011).

YS hematopoiesis has long been considered a transient wave devoted to the production of erythrocytes, megakaryocytes, and a few myeloid cells that ensure oxygenation and tissue hemostasis. HSC-derived hematopoiesis was thought to replace YS-derived cells after HSCs migrate to the FL at E10.5 (Palis, 2016). Recently, growing evidence endows the YS with the capacity to contribute to tissue-resident cells such as macrophages that persist throughout life (Gomez Perdiguero et al., 2015) and mast cells (Gentek et al., 2018) maintained up until birth. Primitive erythrocytes were also shown to persist throughout gestation (Fraser et al., 2007), and EMP-derived cells contribute to the erythrocyte compartment for more than 20 d upon transplantation (McGrath et al., 2015a). Nonetheless, it has been difficult to establish the temporal relative contribution of EMP- or HSC-derived progenitors to erythropoiesis because they share surface markers and transcriptional regulators and are therefore indistinguishable.

Here we report a large population of Kit^+^CD45^−^Ter119^−^ erythroid progenitors unique to FL, comprising >70% of E14.5 Ter119^−^CD45^−^ cells (>10% of FL cells). These are the most actively proliferating progenitors at early stages and rapidly progress in erythroid differentiation. These cells, which require *c-Myb* expression, originate from YS EMPs as they are colabeled with microglia in the Cdh5^CreERT2^ Rosa26^YFP^ and labeled in the Csf1r^MeriCreMer^Rosa26^YFP^ lineage-tracer models induced at E7.5 and at E8.5, respectively. They persist through fetal life and are the major contributors to the RBC compartment.

We show that HSCs do not contribute significantly to embryonic erythropoiesis by tracing Flt3^+^ progenitors or YFP-expressing cells in Cdh5^CreERT2^ Rosa26^YFP^ induced in E10.5 embryos. HSC-derived erythroid progenitors require >10-fold higher concentrations of erythropoietin (Epo) than their YS-derived counterparts for erythrocyte differentiation. The limiting amounts of Epo available in the embryo (Suzuki et al., 2011) result in a selective advantage of YS-derived over HSC-derived erythropoiesis.

## Results

### A unique population of Kit^+^ cells represents the majority of FL Ter119^−^CD45^−^ cells

**Figure 1.**
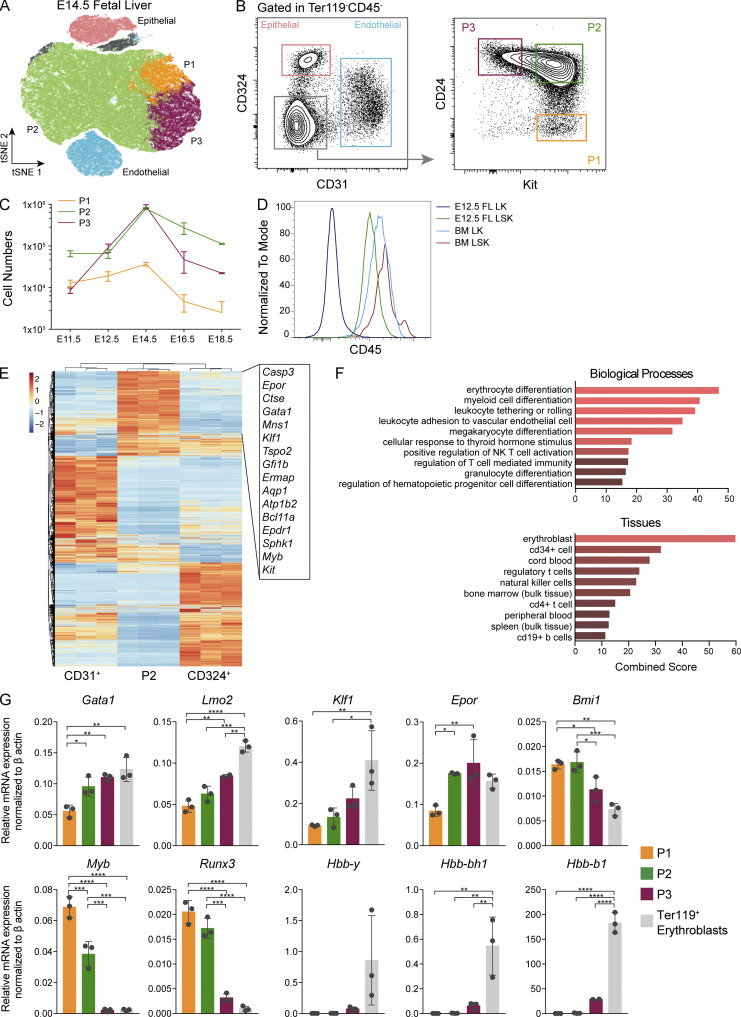
**A population of Kit^+^ cells unique to FL represents the majority of Ter119^−^CD45^−^ cells and has an erythroid progenitor transcriptional signature. (A)** tSNE analysis and hierarchical clustering of flow cytometry data of Ter119^−^CD45^−^ cells from E14.5 FLs stained with the surface markers CD31 (endothelial cells), CD324 (epithelial cells, hepatoblasts), Kit, and CD24. **(B)** Representative FACS plots of the clusters identified in A using the same color code. **(C)** TER119^−^CD45^−^ cell numbers from E12.5 and E18.5. **(D)** Histogram of CD45 expression in LK and LSK cells from E12.5 FL and adult BM (E11.5, *n* = 6; E12.5, *n* = 17; E14.5, *n* = 4; E16.5, *n* = 8; and E18.5, *n* = 2). See also Fig. S1. **(E)** CD31^+^, P2, and CD324 ^+^ cells from E14.5 FL were sorted and subjected to RNA-seq. Differentially expressed genes are represented as a heatmap, and most expressed genes are listed (*n* = 3 independent litters). **(F)** Gene set enrichment analysis on genes with more than twofold difference in expression level between P2 and CD324^+^ cells using Enrichr web application; top 10 significantly associated Gene Ontology Biological Process and ARCHS4 Tissues are shown. The top biological process is erythrocyte differentiation (q-value <1.0e^−6^; Gene Ontology term GO:0030218), and the top tissue associated is erythroblast (q-value <1.0e^−14^; ARCHS4 Tissues gene database). **(G)** E14.5 FL P1, P2, P3, and Ter119^+^ cells were sorted and gene expression of key erythroid genes (*Gata1*, *Lmo2*, *Klf1*, *Epor*, and *Bmi1*), progenitor-associated genes (*c-Myb* and *Runx3*), and hemoglobins (*Hbb-y*, *Hbb-bh1*, and *Hbb-b1*) was analyzed. qPCR data were analyzed using the ΔCt method and were normalized with β-actin. Statistical significance was assessed using one-way ANOVA followed by Tukey’s multiple comparison test. *, P < 0.05; **, P < 0.01; ***, P < 0.001; ****, P < 0.0001. Data are represented as mean ± SD from three independent experiments. NK, natural killer.

**Figure S1.**
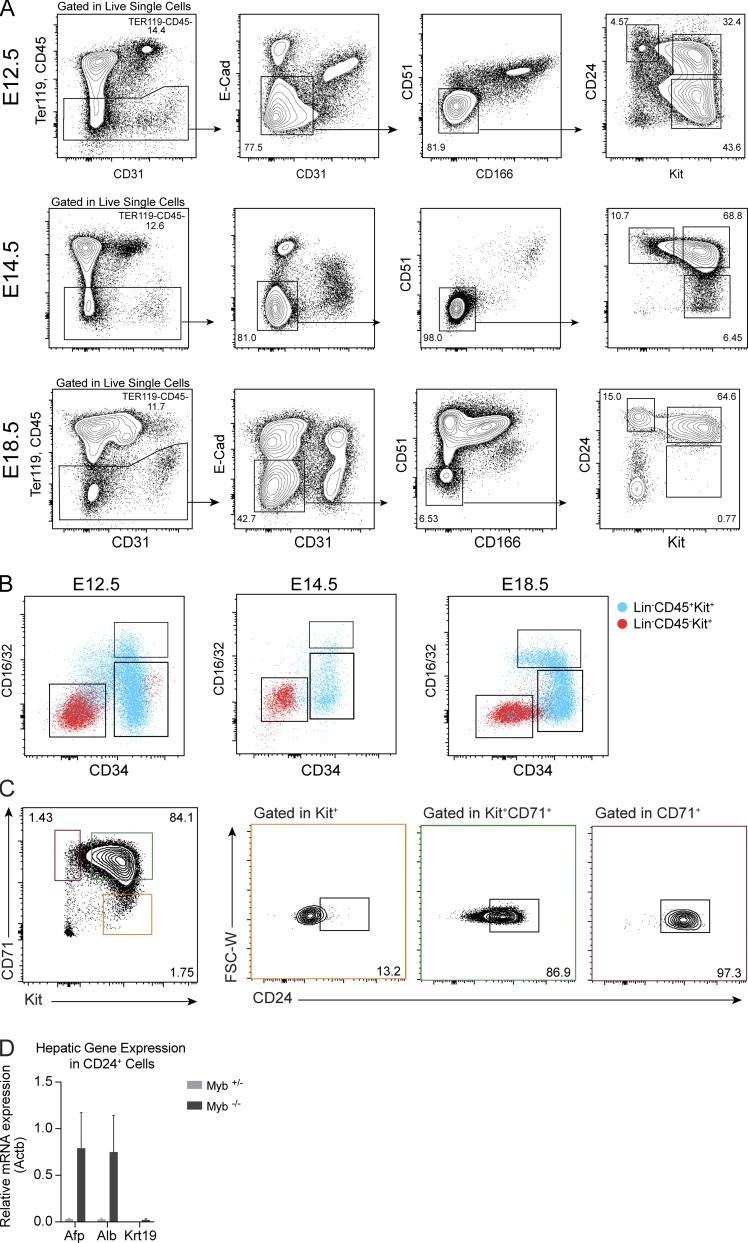
**Phenotype of E12.5, E14.5, and E18.5 FL and adult BM populations (related to ****Figs. 1**** and ****2****). (A)** Flow cytometry analysis of E12.5, E14.5, and E18.5 FL cells using Ter119, CD45, epethilial cadherin (E-cad), CD31, CD51, and CD166. Viable Ter119^−^CD45^−^E-Cad^−^CD31^−^CD51^−^CD166^−^ cells can be subdivided into three populations according to expression of CD24 and Kit. **(B)** Flow cytometry profile of E12.5, E14.5, and E18.5 FL Lin^−^CD45^+^Kit^+^ (CD45^+^LK; blue) and CD45^−^Kit^+^ (red) cells according to CD16/32 and CD34 expression. **(C)** Flow cytometry analysis of CD24 expression in P3 (CD71^+^Kit^−^), P2 (CD71^+^Kit^+^), and P1 (CD71^−^Kit^+^) cells in E13.5 FL cells. **(D)** *Afp*, *Alb*,**and *Krt19* mRNA expression in E14.5 in P2/P3 cells of Myb^+/−^ and Myb^−/−^ FL cells. FSC-W, forward-scatter width.

We detected in FL, by flow cytometry, a large fraction of Kit^+^ cells (>50%) expressing neither Ter119 (specific for erythrocytes) nor CD45 (a pan-hematopoietic marker; Fig. 1, A and B; and Fig. S1). Single-cell surface marker expression data from E14.5 FL cells was projected as tSNE1 vs. tSNE2 (t-distributed stochastic neighbor embedding; Fig. 1 A), and three major clusters were defined by the expression of epithelial cadherin (CD324) on epithelial cells, platelet/endothelial cell adhesion protein (PECAM-1/CD31) on endothelial cells, and Kit. Combined analysis of Kit expression together with CD24 further defined three populations in the Ter119^−^CD45^−^CD31^−^CD324^−^ compartment: Kit^+^CD24^−^ (hereafter called P1), Kit^+^CD24^+^ (P2), and CD24^+^Kit^−^ (P3) cells (Fig. 1 B and Fig. S1 A). Numbers of P2 cells reached a maximum (around 10^6^ cells per FL) at E14–E15 and decreased thereafter, although they were still detected around birth (E18.5; Fig. 1 C). Kit^+^CD45^−^Ter119^−^Lin^−^ (P1 and P2) cells were also negative for the expression of Sca-1, which marks multipotent progenitors, for CD16/32, which marks granulocyte/macrophage progenitors (GMPs), and for CD34, marking common myeloid progenitors (CMPs), and therefore they fall in a gate that typically defines MEPs in the FL and in the BM (Fig. S1 B). Unlike their BM counterparts, however, where all Kit^+^ cells coexpressed CD45, most FL Kit^+^ (P1 and P2) cells within the Lin^−^Kit^+^Sca1^−^ (LK) compartment did not express CD45 (Fig. 1 D), raising the possibility that they did not belong to the hematopoietic lineage. We identified here a major population of Kit^+^CD45^−^Ter119^−^ cells unique to FL of undefined lineage affiliation and origin.

### P1 and P2 cells in the FL have an erythroid progenitor signature

RNA sequencing (RNA-seq) of the three major populations P2, CD324^+^, and CD31^+^ cells indicated that the highest expressed transcripts in P2 cells were *Myb*, *Bcl11a*, *Klf1*, *Gata1*, and *Epor*, which are associated with erythrocyte differentiation (Fig. 1 E). The 122 genes upregulated more than twofold in P2 vs. CD324^+^ cells were subjected to gene ontology analysis using Enrichr (Chen et al., 2013; list of submitted genes in Table S1). The top biological processes and tissue-associated genes revealed an erythrocyte/erythroblast profile (Fig. 1 F). These results were validated by quantitative RT-PCR indicating that *Gata1*, *Lmo2*, *Klf1*, and *Epor* expressions gradually increased from P1 to P3 cells, with the latter showing comparable expression levels of these transcripts to Ter119^+^ erythroblasts (Fig. 1 G). Hemoglobin transcripts for *Hbb-y*, *Hbb-bh1*, and *Hbb-b1* were detected in P3 cells but only significantly expressed in Ter119^+^ cells. Multipotent hematopoiesis associated transcription factors such as *c-Myb*, *Runx3*, and *Bmi1* decreased as the erythroid-specific transcripts increased. The results above indicated that the CD45^−^Kit^+^ subsets (P1 and P2) are erythroid progenitors and suggested a hierarchy where P1 cells further differentiate into P2 and later lose Kit expression (P3) before acquiring Ter119 expression, the definitive marker of erythroid identity.

### P1, P2, and P3 cells represent increasingly mature stages within the erythroid lineage

**Figure 2.**
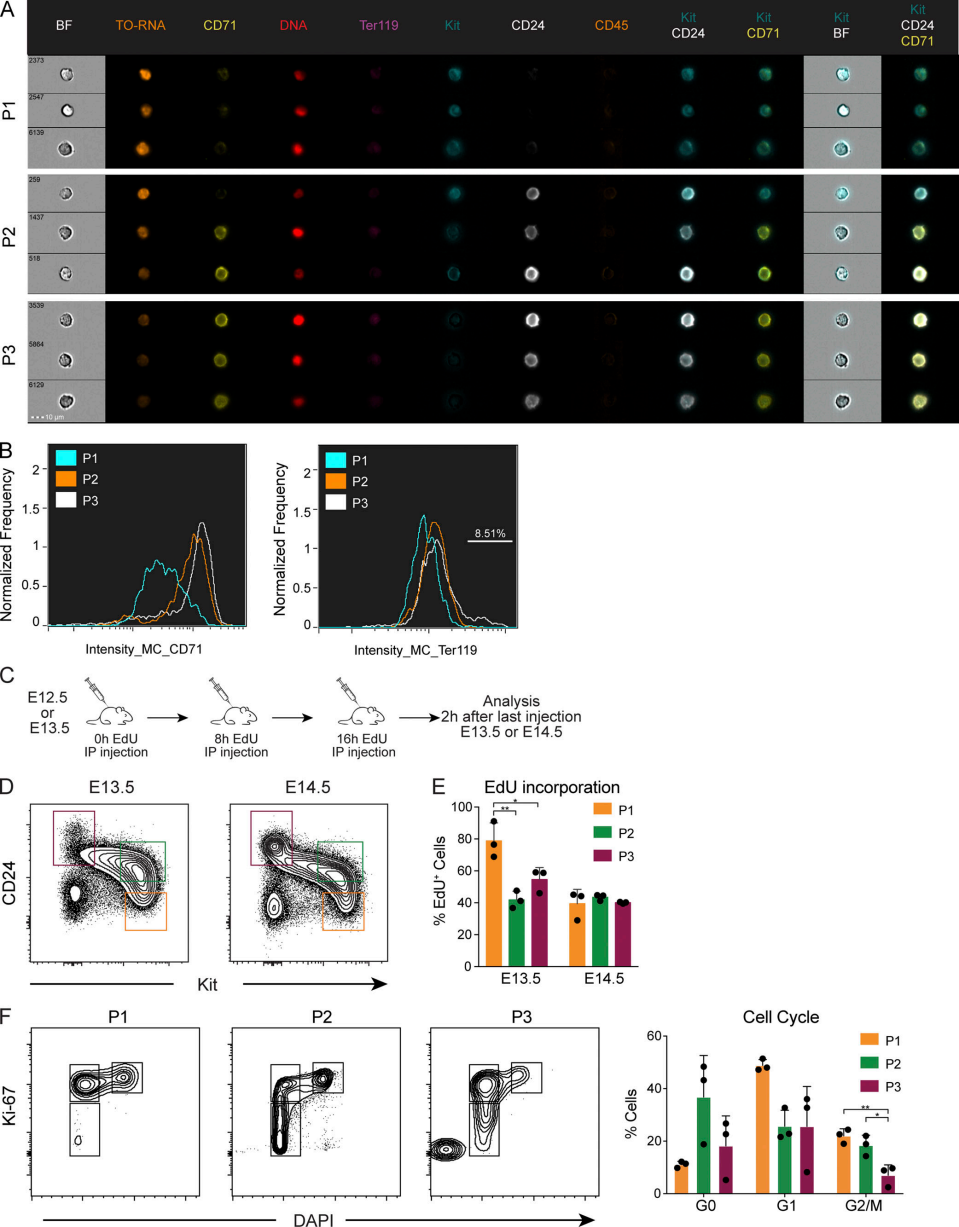
**P1, P2, and P3 cells represent increasingly mature stages within the erythroid lineage. (A)** E13.5 Ter119^−^CD45^−^ cells were analyzed by imaging flow cytometry using CD71, Ter119, Kit, CD24, and CD45 as surface markers, DRAQ5 to label nuclei, and Thiazole Orange to label RNA. Representative images of P1, P2, and P3 cells. BF, Brightfield. **(B)** Expression of CD71 and Ter119 was assessed in P1, P2, and P3 cells and plotted as a histogram. **(C and D)** Experimental design of cell cycle analysis using EdU labeling. E12.5 or E13.5 pregnant mice were injected intraperitoneally (IP) with 100 μg EdU at 0 h, 8 h, and 16 h. FLs were collected 2 h after the last injection, and EdU expression was analyzed on Ter119^−^CD45^−^CD54^−^CD31^−^ cells using the indicated gates (D).** (E)** Percentages of EdU incorporation in P1, P2, and P3 cells at E13.5 and E14.5 (*n* = 3). **(F)** Cell cycle analysis of E14.5 FL cells using Ki-67 and DAPI. Statistical significance was assessed using one-way ANOVA followed by Tukey’s multiple comparison test. *, P < 0.05; **, P < 0.01. Data are represented as mean ± SD from three independent experiments.

Erythroid differentiation has been characterized by the expression of CD71 (transferrin receptor) and CD44 in Ter119^+^ cells (McGrath et al., 2017). Imaging flow cytometry (Fig. 2 A) showed that the pro-erythroblast marker CD71 was low in P1, increased in P2, and was highly expressed in all P3 cells (Fig. 2 B), indicating they correspond to consecutive stages of erythrocyte development.

In BM and FL, CFU-Es are characterized as Kit^+^CD71^+^Ter119^−^, whereas low levels of Ter119 expression mark pro-erythroblasts that lost proliferative capacity (McGrath et al., 2017). P2 FL cells express CD71 but not Ter119, indicating that they correspond to CFU-Es. P3 cells express low levels of Ter119 (Fig. 2 B), visible in 8% of them, and express four out of the five key erythroid genes analyzed at levels similar to Ter119^+^ erythroblasts (Fig. 1 G), suggesting they correspond to proerythroblasts.

CD71 expression is limited to P2 and P3 cells, indicating that CD71 and CD24 are redundant markers in this context, further confirmed by conventional flow cytometry, as previously described (Fig. S1 C; Flygare et al., 2011).

Administration of three doses of the nucleotide analogue 5-ethynyl-2′-deoxyuridine (EdU), which labels newly synthesized DNA, in E12.5 or E13.5 pregnant females (Fig. 2 C) indicated that P1 E13.5 FL cells are the highest proliferating cells (80% EdU^+^) compared with P2 and P3 cells (∼50% EdU^+^). E14.5 FL cells show the same level of EdU incorporation (40–50%) in all three subsets (Fig. 2, D and E). Cell proliferation was further assessed by analyzing the expression of the nuclear protein Ki-67 that in association with DAPI allows distinguishing cells in G0, G1, and G2M phases of the cell cycle. Consistent with the EdU-labeling experiments, P1 showed the lowest frequency of cells in G0 (∼10%) and the highest frequency of cells expressing Ki-67, from which around 20% were actively synthesizing DNA (DAPI^+^; Fig. 2 F). By contrast, P3 cells showed the lowest frequency of proliferating cells (Fig. 2 F). Taken together, these results indicated that P1 are the most proliferating cells and, as they transit onto the P2 and further into the P3 subset, lose proliferative activity.

### P2 and P3 cells are committed erythroid progenitors, whereas P1 cells retain residual myeloid potential

**Figure 3.**
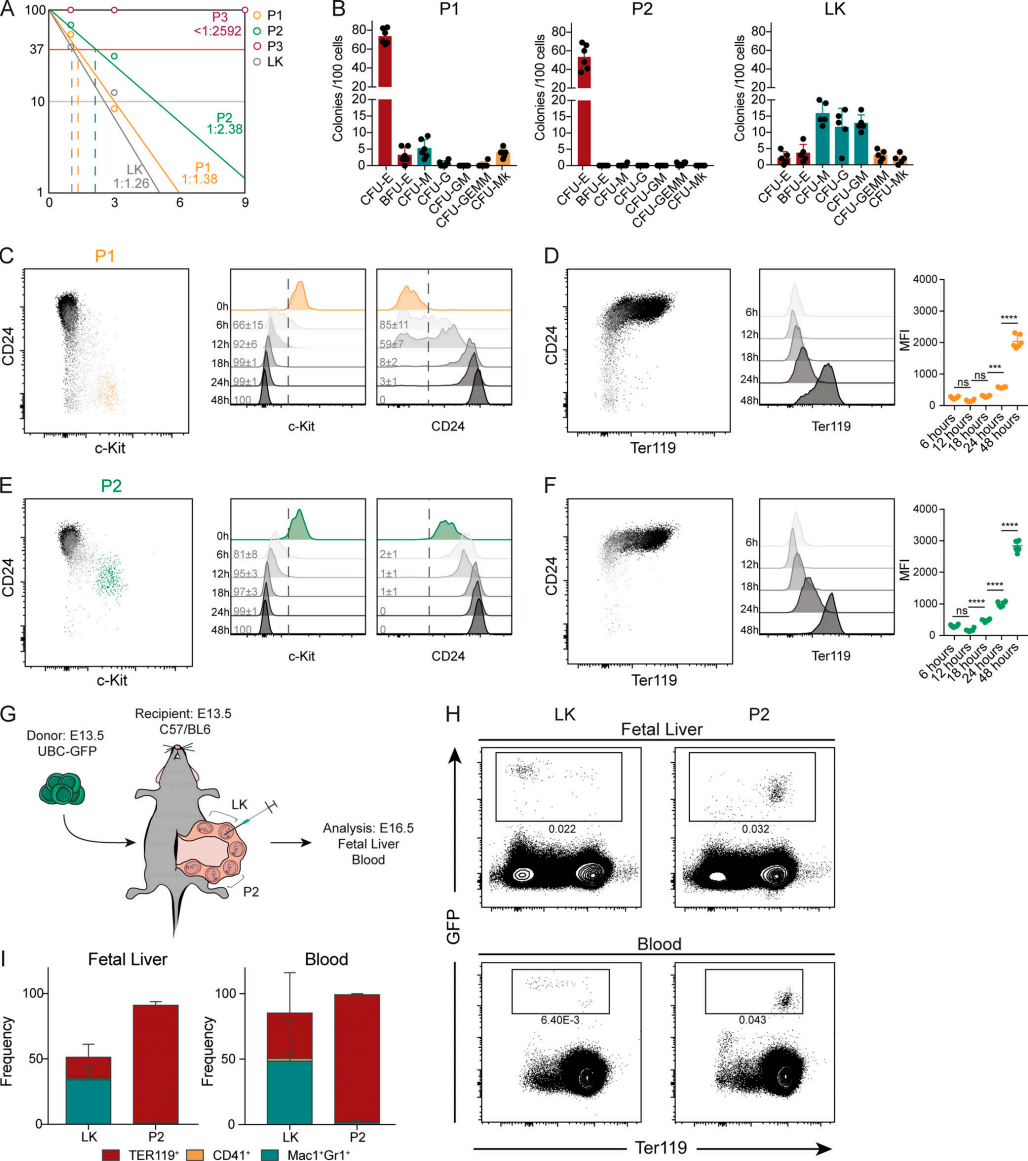
**P2 and P3 cells are committed erythroid progenitors, whereas P1 cells retain residual myeloid potential. (A)** Frequencies of colony forming cells in P1, P2, P3, and LK cells from E13.5 FL (*n* = 288 wells from three independent experiments of each population). **(B)** In vitro lineage potential of E13.5 P1, P2, P3, and LK cells in semi-solid cultures. CFU-E colonies were quantified at 3 d and BFU-E, CFU-M, CFU-G, CFU-GM, CFU-GEMM, and CFU-Mk at 7 d of culture (*n* = 6, three independent experiments). **(C and D) **Representative FACS plots and histograms of CD24 and c-Kit (C) and Ter119 expression and respective mean fluorescence intensity (MFI; D) of P1 cells after culture. **(E and F)** Representative FACS plots and histograms of CD24 and c-Kit (E) and Ter119 expression and respective MFI (F) of P1 cells after culture (*n* = 6, three independent experiments). **(G)** Schematic representation of transplantation experiment. E13.5 C57/BL6 pregnant females were anesthetized, the peritoneal cavity was opened, and the uterus was exposed. Embryos were injected intraperitoneally with 20,000 E13.5 GFP^+^ purified cells from UBC-GFP embryos. FL and blood were collected 3 d after injection. **(H)** Representative FACS plots showing erythroid contribution of GFP^+^ cells in FL and blood after injection of P2 or LK cells and quantification (I; P2 *n* = 3, LK *n* = 4, four independent experiments). Statistical significance was assessed using one-way ANOVA followed by Tukey’s multiple comparison test. ***, P < 0.001; ****, P < 0.0001. Data are represented as mean ± SD.

To assess the differentiation potential of CD45^−^Kit^+^ FL cells, we performed differentiation assays in liquid cultures and in semi-solid colony assays (Fig. 3, A and B). Limiting-dilution analysis showed that P1 and P2 gave rise to hematopoietic colonies at frequencies similar to those of LKs (1:1 for LK and P1, 1:2 for P2 cells), while P3 cells did not divide significantly in culture (less than one colony in 2,592 wells analyzed; Fig. 3 A). Both P1 and P2 cells generated a majority of CFU-Es (>50% of plated cells) in semi-solid colony assays. P1 cells also generated BFU-Es (<5%), CFU-macrophages (CFU-Ms; 5%), and CFU-megakaryocytes (CFU-Mks; 5%), whereas control (LK) generated a majority of myeloid colonies (CFU-granulocytes [CFU-Gs], CFU-granulocyte/macrophages [CFU-GMs], and CFU–granulocyte/erythroid/macrophage/megakaryocytes [CFU-GEMMs]; Fig. 3 B).

To show a lineage relationship between P1, P2, and P3 cells, we cultured purified P1 and P2 cells. 6 h in culture was sufficient to upregulate CD24 expression in P1 cells, and after 12 h, 40% of cells expressed CD24 (Fig. 3 C). After 24 h, all P1 cells had differentiated to P2 cells, upregulating CD24 and losing c-Kit expression (Fig. 3 C). Ter119 expression was detected in P1 cells after 48 h in culture, whereas P2 cells already expressed this marker after 18 h of culture, demonstrating that they represent a more differentiated progenitor (Fig. 3, D and F).

To probe the differentiation potential in vivo, we injected P2 cells purified from E13.5 ubiquitin C (UBC)–GFP embryos into E13.5 C57/BL6 recipient embryos in utero (Fig. 3 G). FL and blood collected 3 d later indicated that GFP^+^P2 originated exclusively GFP^+^Ter119^+^ cells whereas LKs generated a majority of myeloid cells (Fig. 3, H and I), while none gave rise to lymphocytes. The low numbers of P1 cells precluded similar in vivo differentiation analysis with these cells.

These results demonstrated that P2 FL cells are committed erythroid progenitors while P1 cells retain residual in vitro myeloid differentiation potential.

### P1/P2 progenitors require *c-Myb* expression

**Figure 4.**
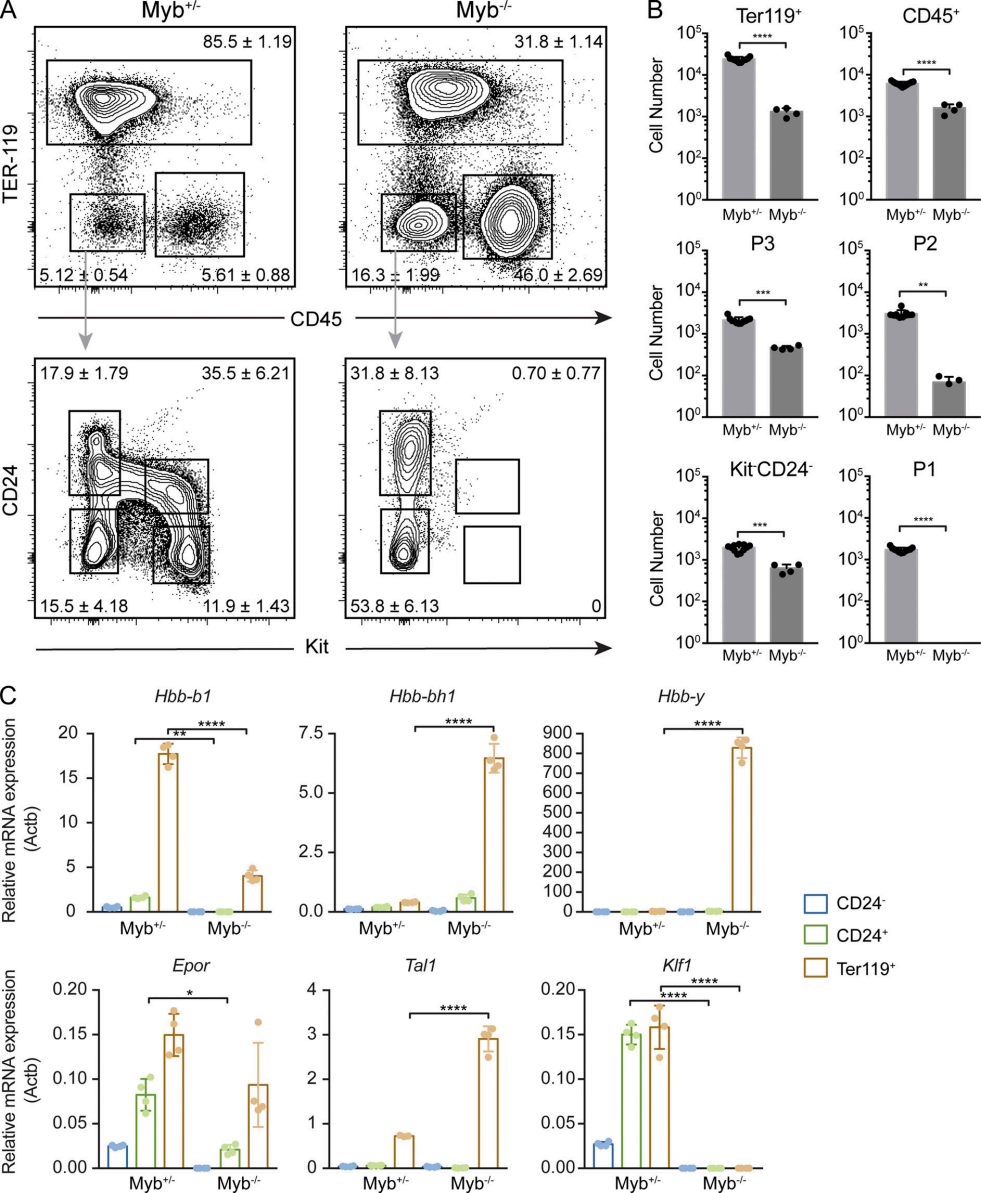
**P1/P2 progenitors require *c-Myb* expression. (A and B)** Representative FACS plots showing percentages of Ter119 and CD45 (top) and Kit and CD24 (bottom) expressing cells in c-Myb^+/−^ and c-Myb^−/−^ E14.5 FL and corresponding absolute numbers (B).** (C)** Expression of hemoglobin (*Hbb-b1*, *Hbb-bh1*, and *Hbb-y*) and key erythroid genes (*Epor*, *Tal1*, and *Klf1*) in CD24^−^, CD24^+^, and Ter119^+^ cells was analyzed by qPCR. Statistical significance was assessed using one-way ANOVA followed by Tukey’s multiple comparison test. *, P < 0.05; **, P < 0.01; ***, P < 0.001; ****, P < 0.0001. Data are represented as mean ± SD from two independent experiments.

The transcription factor *c-Myb* is essential for definitive hematopoiesis, and Myb*^−/−^* embryos are not viable after E15. Only primitive YS-derived erythropoiesis, primitive megakaryocytes (Tober et al., 2008), and tissue-resident macrophages (Schulz et al., 2012) are found in *c-Myb* mutants. Ter119^+^ cells were drastically decreased in frequency and numbers in Myb^−/−^ FLs when compared with heterozygous littermates (Fig. 4, A and B). P1 cells were undetectable in Myb^−/−^ whereas P3 cells were present, albeit in reduced numbers (Fig. 4 B). CD24^+^ cells in Myb^−/−^ FL expressed high levels of *Afp* and *Alb*, indicating their hepatic cell affiliation (Fig. S1 D). *c-Myb* was reported to regulate c-Kit expression (Ratajczak et al., 1998). To assess whether P1 and P2 cells, although unable to express c-Kit, were present in the Myb^−/−^ FL, we analyzed the expression of erythroid genes in CD24^−^, CD24^+^, and Ter119^+^ cells from Myb^+/−^ and Myb^−/−^ FLs. *Epor*, *Tal1*, and *Klf1* were not detected in CD24^−^ and CD24^+^Myb^−/−^ compared with Myb^+/−^ cells (Fig. 4 C). Only Ter119^+^ cells expressed detectable levels of *Epor* and *Tal1* together with high levels of *Hbb-y*, indicating they represent primitive erythrocytes. Of note, *Klf1*, a transcription activator of the β-globin promoter, was not expressed in primitive Ter119^+^Myb^−/−^ cells (Fig. 4 C). These results demonstrated that differentiation and/or survival of CD45^−^Kit^+^ erythroid progenitors required the transcription factor *c-Myb*.

### P1/P2 cells originate from YS progenitors and are major contributors to embryonic erythropoiesis

**Figure 5.**
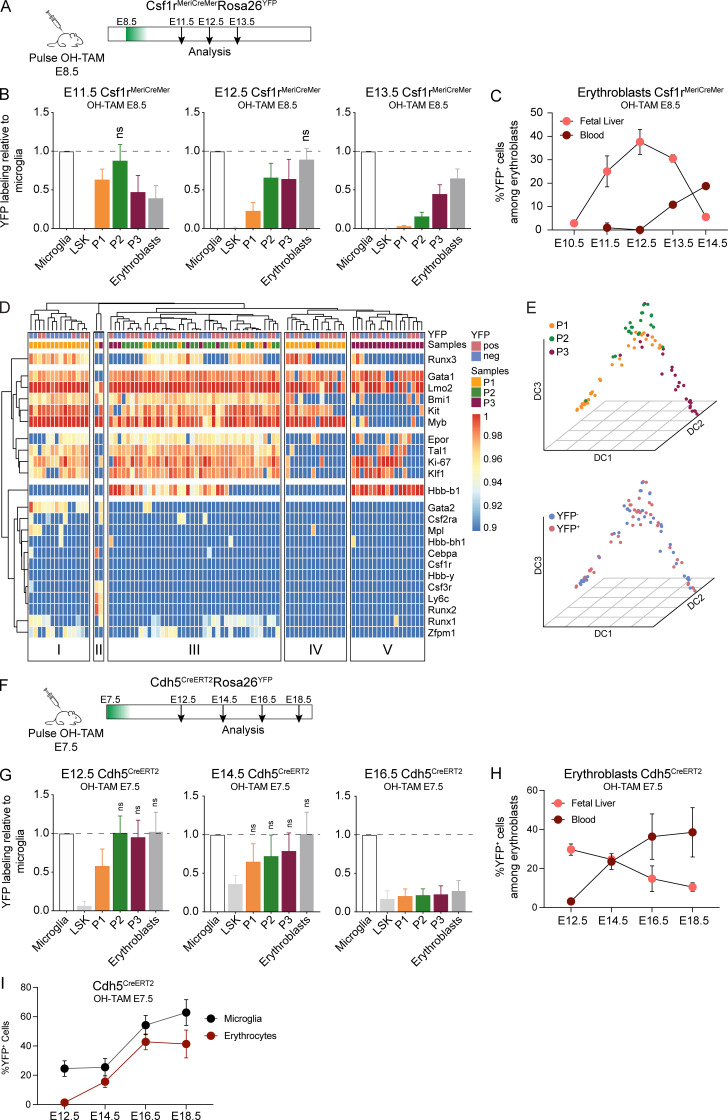
**P1/P2 cells originate from YS and are major contributors to embryonic erythropoiesis. (A)** Experimental design for lineage tracing experiments using the Csf1r^MeriCreMer^Rosa26^YFP^. Cre recombinase expression was induced in Csf1r^MeriCreMer^Rosa26^YFP^ pregnant females with a single injection of OH-TAM at E8.5, and embryos were analyzed 3, 4, and 5 d after injection. **(B)** Ratio of YFP^+^ LSKs and the different erythroid subsets over that in microglia at E11.5, E12.5, and E13.5 after a pulse of OH-TAM at E8.5 (E11.5, *n* = 12; E12.5, *n* = 11; and E13.5, *n* = 7). **(C)** Frequency of YFP^+^ erythroblasts in FL (pink) and blood (red) from E10.5 to E14.5 after a pulse of OH-TAM at E8.5 (E10.5, *n* = 3; E11.5, *n* = 12; E12.5, *n* = 11; E13.5, *n* = 7; and E14.5, *n* = 2). Only nonstatistically different results are noted. **(D)** Heatmap of single-cell qPCR in sorted cells from Csf1r^MeriCreMer^ E13.5 FL pulsed with OH-TAM at E8.5. Each column represents a single cell, and it is color-coded according to YFP expression (YFP^+^ vs. YFP^−^) and cell type (P1, P2, or P3). Gene expression was normalized to *β-actin* and *Gapdh*, and unsupervised hierarchical clustering was performed. **(E)** Diffusion map of the three populations based on single-cell gene expression. Top: Color-coding according to cell type. Bottom: Color-coding according to YFP expression. A total of 87 cells were analyzed. **(F)** Experimental design for lineage tracing experiments using the Cdh5^CreERT2^Rosa26^YFP^. Cre recombinase expression was induced in Cdh5^CreERT2^Rosa26^YFP^ pregnant females with a single injection of OH-TAM at E7.5, and embryos were analyzed 5, 7, and 9 d after injection. **(G)** Ratio of YFP^+^ LSK and erythroid cells over those in microglia at E12.5, E14.5, and E16.5 after a pulse of OH-TAM at E7.5 (E12.5, *n* = 4; E14.5, *n* = 5; and E16.5, *n* = 6). Only nonstatistically different results are noted. **(H)** Percentage of YFP^+^ erythroblasts in FL (pink) and blood (red) at E12.5, E14.5, and E16.5 after a pulse of OH-TAM at E7.5. **(I)** YFP^+^ microglia and blood erythrocytes at E12.5, E14.5, E16.5, and E18.5 after a pulse of OH-TAM at E7.5. Microglia (CD45^+^F4/80^+^CD11b^+^) cells were used as controls (E12.5, *n* = 4; E14.5, *n* = 5; E16.5, *n* = 11; and E18.5, *n* = 4). Statistical significance was assessed using one-way ANOVA followed by Tukey’s multiple comparison test. Data are represented as mean ± SD. See also Figs. S2 and S3. neg, negative; pos, positive.

**Figure S2.**
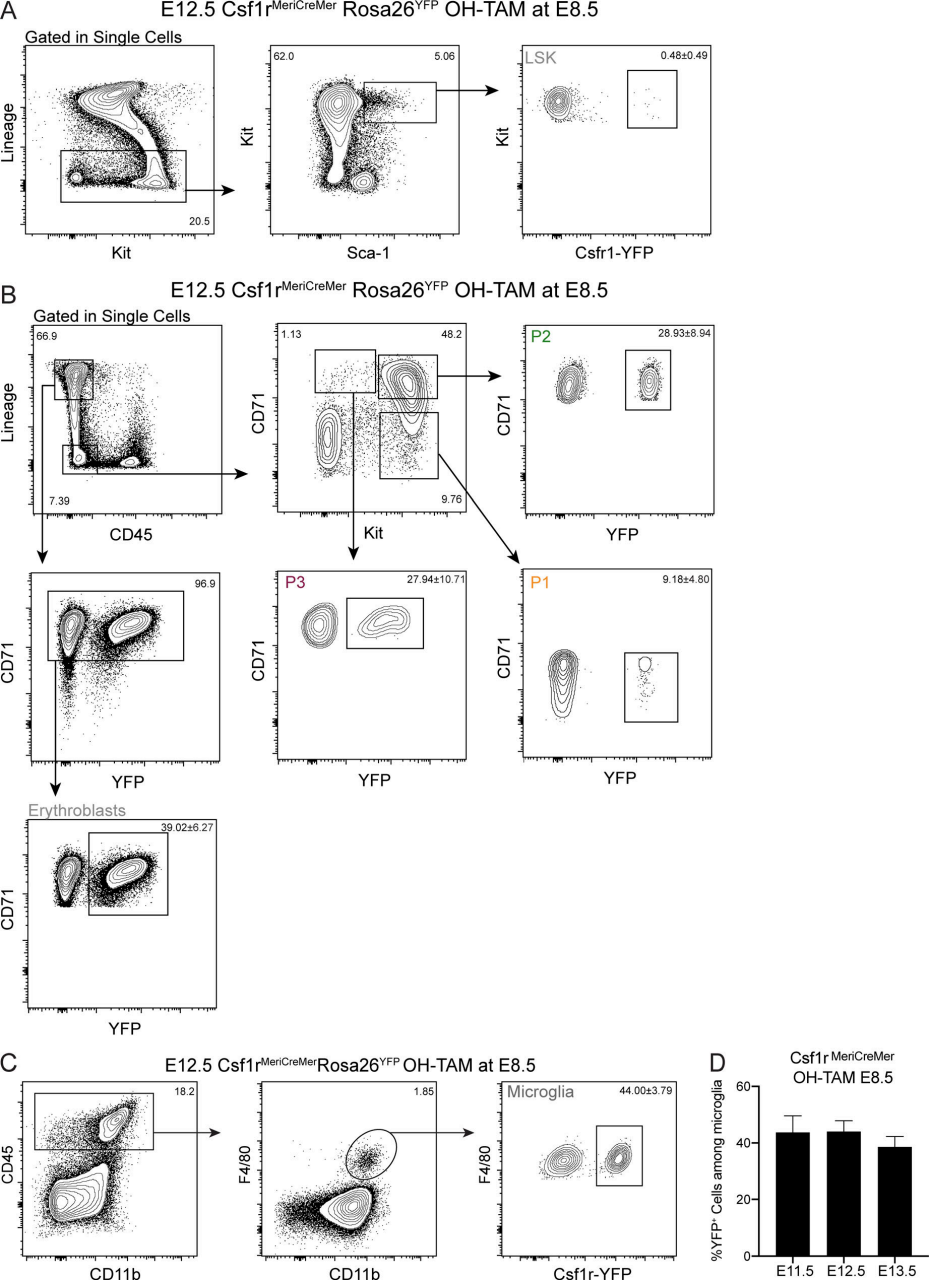
**Gating strategies used to determine YFP-labeled hematopoietic populations in the Csf1r^MeriCreMer^Rosa26^YFP^ lineage tracing model (related to ****Fig. 5****).** **(A)** Gating strategy for the analysis of YFP expression in LSK cells. **(B)** Gating strategy for the analysis of YFP expression in P1 (Lin^−^CD45^−^Kit^+^CD71^−^), P2 (Lin^−^CD45^−^Kit^+^CD71^+^), P3 (Lin^−^CD45^−^Kit^−^CD71^+^), and erythroblast (Lin^+^CD71^+^) cells. **(C)** Gating strategy for the analysis of YFP expression in microglia (CD45^+^F4/80^+^CD11b^+^) cells. Data representative of E12.5 YFP^+^ embryos. **(D)** Percentage of YFP^+^ cells among microglia at E11.5, E12.5, and E13.5.

P1/P2 (CD45^−^Kit^+^) cells were not detected in the adult BM, suggesting that they represent a transient hematopoietic population. To assess the origin of P1/P2 cells, we analyzed FL from Csf1r^MeriCreMer^Rosa26^YFP^ pregnant females pulsed with a single dose of 4-hydroxytamoxifen (OH-TAM) at E8.5 (Fig. 5 A; and Fig. S2, A and B), which marks tissue resident macrophages but virtually no HSC-derived progenitors (Gomez Perdiguero et al., 2015). At E11.5, P1 and P2 cells were labeled to comparable levels of those in the microglia, taken as positive controls (Fig. 5 B; and Fig. S2, C and D). In subsequent days, the frequency of YFP^+^ cells in more differentiated P3 and erythroblasts (Lin^+^CD71^+^) increased, whereas that of more immature P1 cells decreased. In line with previous reports, YFP^+^ Lin^−^Kit^+^Sca1^+^ cells (LSKs) were undetectable (Fig. 5 B and Fig. S2 A). The dynamic of YFP-labeled erythroid progenitors is consistent with a progression in erythroid differentiation and indicates a lineage relationship between the three subsets. Moreover, the frequency of YFP-labeled erythroblasts decreases in FL from E12.5 until E14.5 while it increases in blood (Fig. 5 C). The frequency of YFP-labeled P1 and P2 decreases between E11.5 and E13.5, a dynamic that reflects a fast differentiation progression, culminating with the exit of erythroblasts from FL into circulation.

To test whether YFP^+^ and YFP^−^ cells represented two distinct populations, we performed single-cell multiplex gene expression analysis in FL P1, P2, and P3 cells of Csf1r^MeriCreMer^Rosa26^YFP^ embryos pulsed at E8.5. Unsupervised hierarchical clustering did not segregate YFP^+^ from YFP^−^ cells for the expression of progenitor, erythroid, and myeloid transcripts, indicating that they have a similar profile and therefore do not represent two divergent progenitor populations (Fig. 5 D). Clusters I and IV contained P1 cells characterized by the expression of *Gata1*, *Lmo2*, and *c-Myb*. Cluster I differed from cluster IV by a high frequency of cells expressing *Epor*, *Tal1*, *Klf1*, and *Ki-67*. Interestingly, some cells in this cluster also coexpressed the myeloid factors *Runx1*, *Gata2*, *Zfpm1*, and *Mpl*, suggesting a broad myeloid transcriptional priming, consistent with data from in vitro differentiation assays (Fig. 4 B). High expression of *Ki-67* also indicates that cluster I in contrast to cluster IV cells are proliferating. Few cells segregated from all others in cluster II, defined by expression of *Csf3r*, *Ly6c*, and *Runx2* in the absence of erythroid-associated transcripts. Cluster III comprises a majority of P2 cells, expressing high levels of erythroid genes and low levels of hemoglobin, thus defining a transitional erythroid population. Cluster V contains P3 cells that express high levels of hemoglobin in the absence of *c-Kit* or *c-Myb*.

To analyze the differentiation trajectory between the three populations, we generated a diffusion map and obtained a trajectory in which P1 cells progress through a P2 and subsequently a P3 stage (Fig. 5 E). This differentiation trajectory is in line with the gene expression data (Fig. 1 G), with the imaging flow cytometry results (Fig. 2 A) and with the clonal differentiation assays (Fig. 3, B–F). YFP^+^ and YFP^−^ cells have similar trajectories, indicating they do not represent different progenitor subsets (Fig. 5 E).

**Figure S3.**
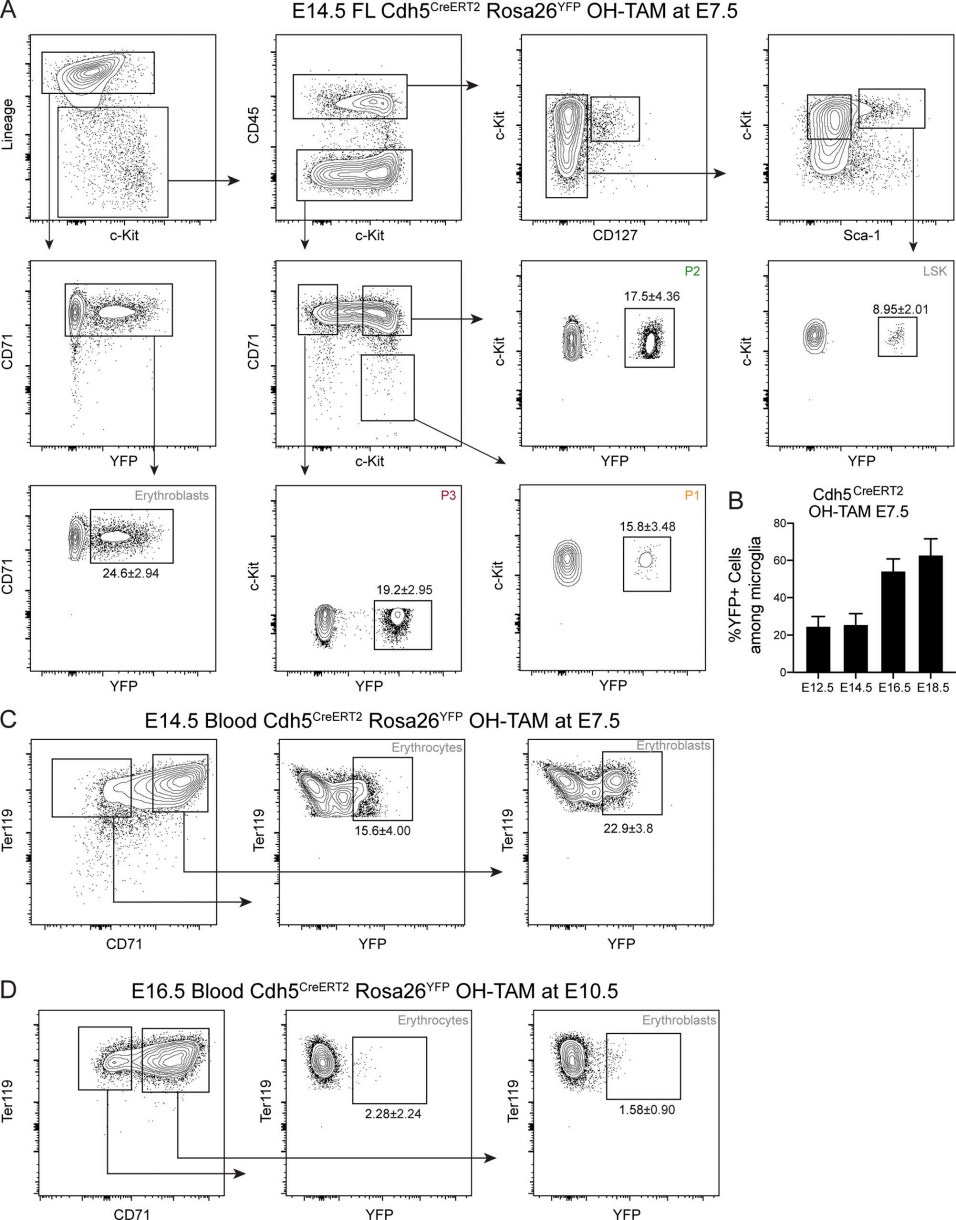
**Gating strategies used to determine YFP-labeled hematopoietic populations in the Cdh5^CreERT2^Rosa26^YFP^ fate-mapping model (related to ****Fig. 5****). (A)** Gating strategy for the analysis of YFP expression in LSK, P1 (Lin^−^CD45^−^Kit^+^CD71^−^), P2 (Lin^−^CD45^−^Kit^+^CD71^+^), P3 (Lin^−^CD45^−^Kit^−^CD71^+^), and erythroblast (Lin^+^CD71^+^) cells. Data representative of E14.5 YFP^+^ embryos. **(B)** Percentage of YFP^+^ cells among microglia at E12.5, E14.5, and E16.5. **(C)** Gating strategy for the analysis of YFP expression in blood erythrocytes (Ter119^+^CD71^−^) and erythroblasts (Ter119^+^CD71^+^) after OH-TAM pulse at E7.5. **(D)** Gating strategy for the analysis of YFP expression in blood erythrocytes (Ter119^+^CD71^−^) and erythroblasts (Ter119^+^CD71^+^) after OH-TAM pulse at E10.5.

The Csf1r^MeriCreMer^Rosa26^YFP^ lineage-tracing model induced at E8.5 labels Csf1r-expressing EMPs and macrophages. To complement the results above and to minimize the possibility of disregarding the contribution of Csf1r^−^ YS progenitors, we used the Cdh5^CreERT2^Rosa26^YFP^ model that, with a single dose of OH-TAM at E7.5, efficiently labels YS hematopoiesis (Fig. 5 F; and Fig. S3, A and B; Zovein et al., 2008; Gentek et al., 2018). P2, P3 and erythroblasts are labeled at the same frequency as microglia at E12.5 and E14.5 and decrease thereafter (Fig. 5 G), similar to the results obtained with Csf1r^MeriCreMer^Rosa26^YFP^ embryos. While YFP^+^ FL erythroblasts decrease from E12.5 to E18.5, blood erythroblasts increase (Fig. 5 H; and Fig. S3, C and D), indicating that they exit the FL and enter circulation. Moreover, the percentage of YFP^+^ blood erythrocytes is similar to that found in microglia (Fig. 5 I), establishing that circulating erythrocytes of EMP YS origin dominate the compartment up until E18.5.

### HSCs do not contribute to erythropoiesis up until birth

**Figure 6.**
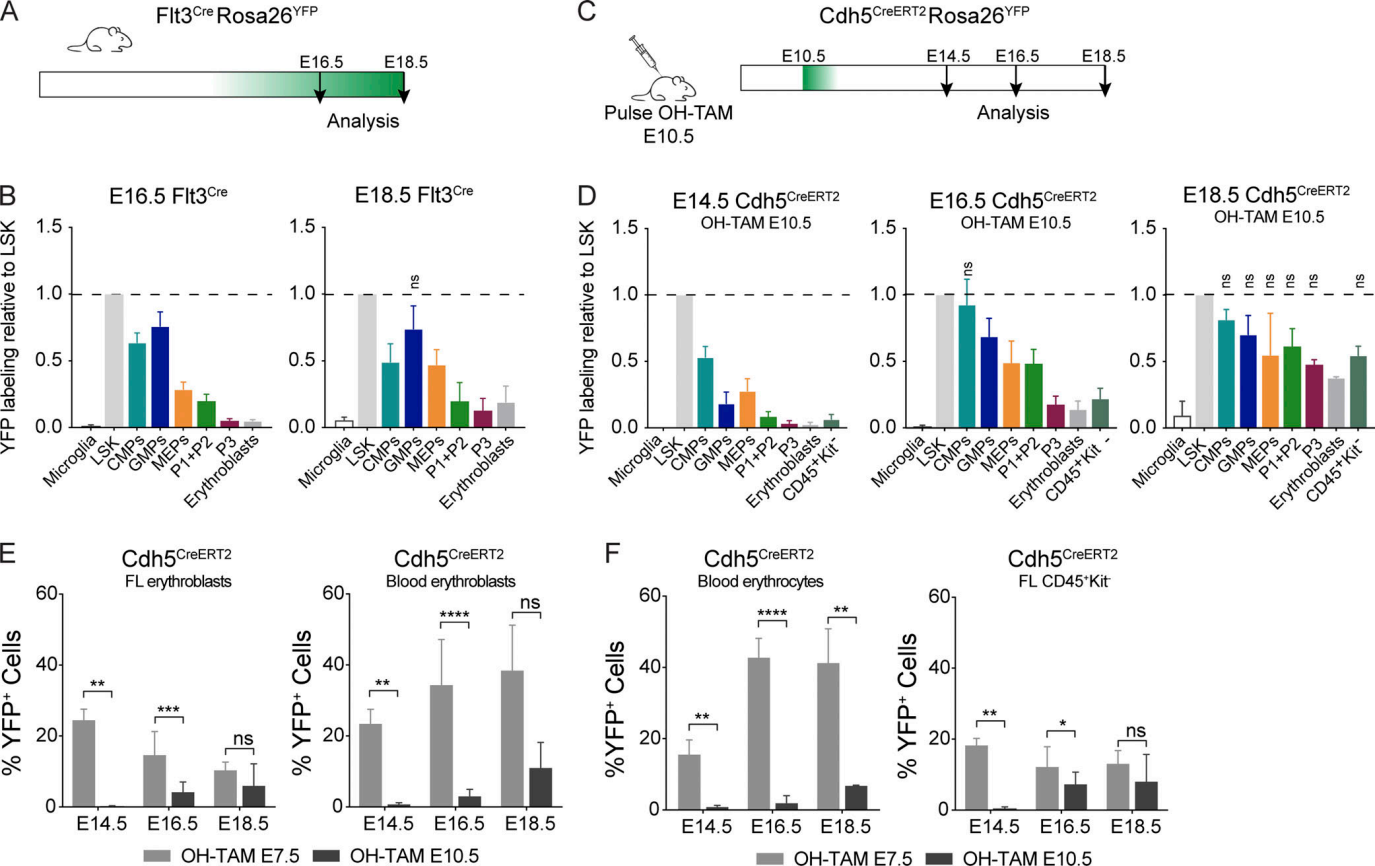
**HSCs do not contribute to erythropoiesis up until birth. (A)** Experimental design for lineage tracing experiments using the Flt3^Cre^Rosa26^YFP^ mice model. Embryos were analyzed at E16.5 and E18.5. **(B)** Ratio of YFP^+^ hematopoietic subsets over those in LSK at E16.5 and E18.5 (E16.5, *n* = 3; and E18.5, *n* = 4). Only nonstatistically different results are noted. **(C)** Experimental design for lineage tracing experiments using the Cdh5^CreERT2^Rosa26^YFP^ mouse model. Cre recombinase expression was induced in Cdh5^CreERT2^Rosa26^YFP^ pregnant females with a single injection of OH-TAM at E10.5, and embryos were analyzed 4, 6, and 8 d after injection. **(D)** Ratio of YFP^+^ hematopoietic subsets over those in LSK at E14.5, E16.5, and E18.5. Only nonstatistically different results are noted. **(E)** Percentage of YFP^+^ erythroblasts in FL (left) and blood (right) at E14.5, E16.5, and E18.5 after a pulse of OH-TAM at E7.5 or at E10.5. **(F)** Percentage of YFP^+^ erythrocytes in blood (left) and CD45^+^Kit^−^ cells in FL (right) at E14.5, E16.5, and E18.5 after a pulse of OH-TAM at E7.5 or at E10.5 (OH-TAM E7.5: E14.5, *n* = 5; E16.5, *n* = 11; and E18.5, *n* = 4; OH-TAM E10.5: E14.5, *n* = 7; E16.5, *n* = 9; and E18.5, *n* = 2). LSK cells were used as controls. Statistical significance was assessed using one-way ANOVA followed by Tukey’s multiple comparison test or Mann–Whitney test when appropriate. *, P < 0.05; **, P < 0.01; ***, P < 0.001; ****, P < 0.0001. Data are represented as mean ± SD. See also Figs. S4 and S5.

**Figure S4.**
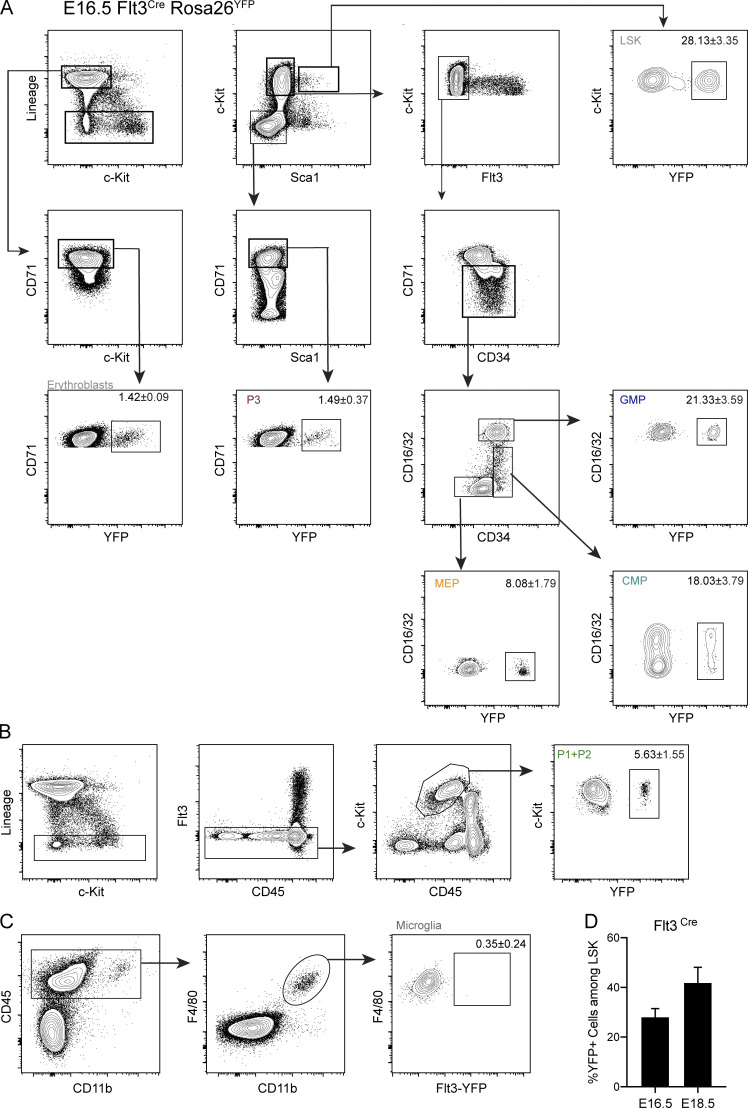
**Gating strategies used to determine YFP labeled hematopoietic populations in the Flt3^Cre^Rosa26^YFP^ fate-mapping model (related to ****Fig. 6****).** **(A)** Gating strategy for the analysis of YFP expression in LSKs, CMPs, GMPs, MEPs, P3 and erythroblasts. **(B)** Gating strategy for the analysis of YFP expression in P1 + P2 cells. **(C)** Gating strategy for the analysis of YFP expression in microglia cells. Data representative of E16.5 YFP^+^ embryos. **(D)** Percentage of YFP^+^ cells among LSKs at E16.5 and E18.5.

**Figure S5.**
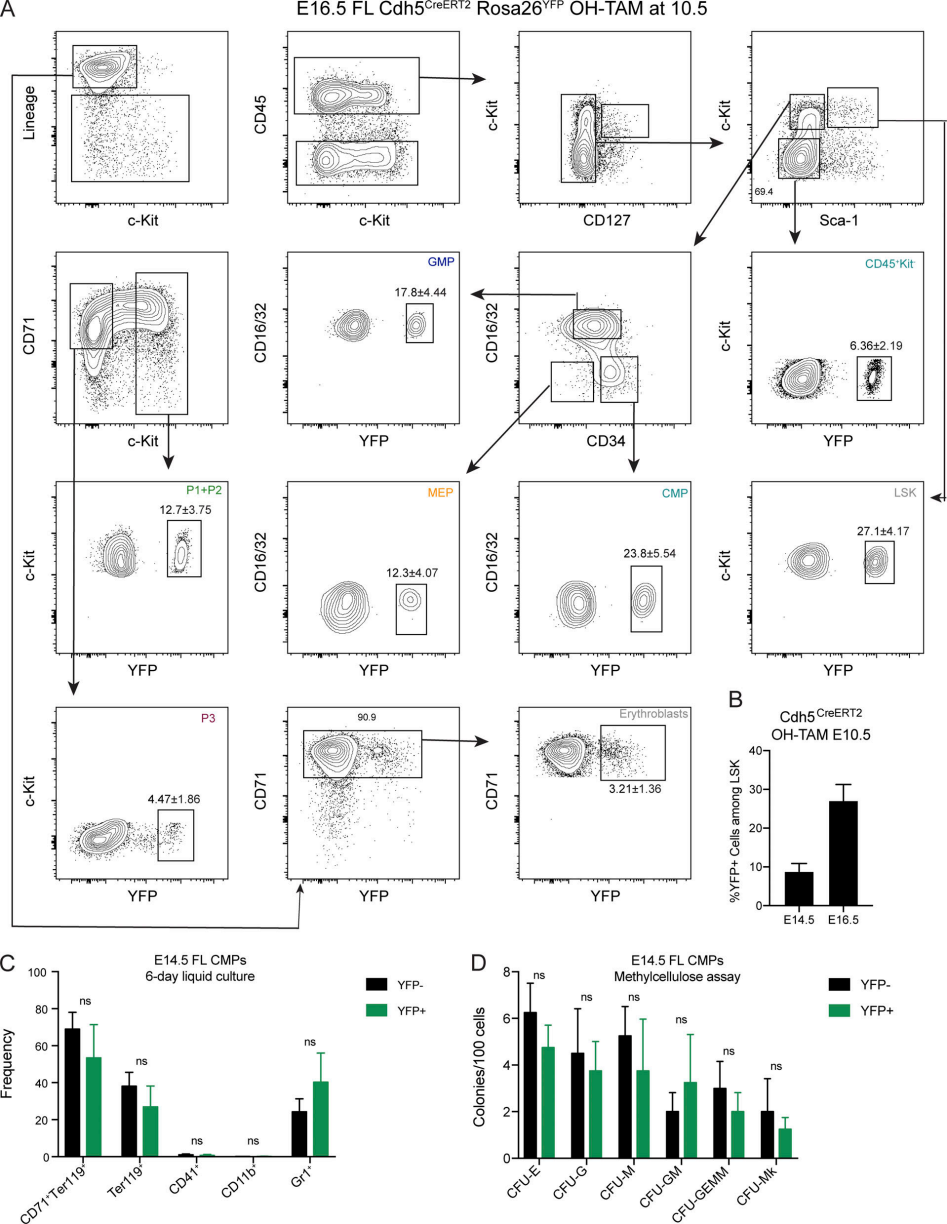
**Gating strategies used to determine YFP labeled hematopoietic populations in the Cdh5^CreERT2^Rosa26^YFP^ fate-mapping model (related to ****Fig. 6****).** **(A)** Gating strategy for the analysis of YFP expression in LSKs, CMPs, GMPs, MEPs, P1 + P2, P3, erythroblasts, and CD45^+^Kit^−^ cells. **(B)** Percentage of YFP^+^ cells among LSKs at E14.5 and E16.5. **(C)** Frequencies of CD71^+^Ter119^+^, Ter119^+^, CD41^+^, CD11b^+^, and Gr1^+^ cells from YFP^−^ or YFP^+^ CMPs from E14.5 FL of Flt3-Cre Rosa26-YFP embryos after 6 d of liquid cultures (YFP^−^, *n* = 10; YFP^+^, *n* = 7; three independent experiments). **(D)** In vitro lineage potential of E14.5 YFP^−^ and YFP^+^ CMPs from Flt3-Cre Rosa26-YFP mice in semi-solid cultures. CFU-E colonies were quantified at 3 d, and CFU-M, CFU-G, CFU-GM, CFU-GEMM, and CFU-Mk at 7 d of culture (*n* = 4, two independent experiments). Statistical significance was assessed using multiple *t* tests. ns, P > 0.05. Data are represented as mean ± SD.

To evaluate the HSC contribution to fetal erythropoiesis, we analyzed Flt3^Cre^Rosa26^YFP^ embryos where HSC-derived multipotent progenitors (MPP Flt3^+^) and their progeny are YFP^+^ (Benz et al., 2008; Buza-Vidas et al., 2011). In addition, it labels to a transient population of Flt3^+^ progenitors recently identified (Fig. 6 A; Beaudin et al., 2016). Less than 2% of microglial cells are YFP^+^ at all time points analyzed. LSKs were increasingly labeled with >30% of YFP^+^ cells at E16.5 and reaching >40% at E18.5 (Fig. S4, A–D). Both CMP and GMP compartments exhibited similar levels of YFP expression to those in LSKs (Fig. 6 B). By contrast, P1 + P2 or P3 cells were minimally labeled with YFP by E18.5. YFP^+^ erythroblasts were virtually undetectable at E16.5 and were <20% of those found in LSKs by E18.5, indicating that HSCs are not contributing to mature erythrocytes up until 1 d before birth. MEPs showed a delayed profile, reaching 50% of LSK labeling only around birth. Purified YFP^−^ and YFP^+^ CMPs yielded similar frequencies of erythroid and myeloid cells and generated similar numbers of hematopoietic colonies, indicating that YFP expression did not impair hematopoietic differentiation (Fig. S5, C and D).

Most erythrocytes are derived from MEPs that differentiate from Flt3-expressing MPPs. However, recent evidence indicated that a fraction of megakaryocytes might bypass the MPP stage and differentiate directly from a progenitor phenotypically indistinguishable from HSCs (Carrelha et al., 2018). These results raised the possibility that erythrocytes may bypass the MPP stage because they originate from a common MEP. Using the Flt3^Cre^Rosa26^YFP^ mouse model, we could therefore underestimate the contribution of HSCs to the erythrocyte compartment. To address this potential problem, we analyzed Cdh5^CreERT2^Rosa26^YFP^ embryos induced at E10.5, thus specifically labeling the aorta-gonads-mesonephros–hemogenic endothelium and all HSC-derived hematopoiesis (Fig. 6 C and Fig. S5 B; Zovein et al., 2008). We obtained similar results with E10.5 induced Cdh5^CreERT2^Rosa26^YFP^ as with Flt3^Cre^Rosa26^YFP^. There was a 1:1 ratio of YFP-labeled CMPs to LSKs at E16.5, in contrast to a 0.5:1 ratio of labeled MEPs or P1 + P2 cells to LSKs and 0.1:1 ratio for erythroblasts. Analysis at E18.5 showed ratios of CMPs, GMPs, MEPs, or P1 + P2 cells over LSKs close to 1:1, while erythroblasts were still <0.5:1 (Fig. 6 D). Further comparison of embryos pulsed with OH-TAM at E7.5 vs. E10.5 showed similar contributions to nonerythroid lineages (CD45^+^Kit^−^ cells) of YS and HSCs at E18.5 (Fig. 6 F), whereas the erythroid compartment is mostly derived from YS hematopoiesis (Fig. 6, E and F). Taken together, these observations indicate that neither HSC-derived nor any other Flt3-expressing progenitor contributes significantly to erythropoiesis throughout fetal life.

**Figure 7.**
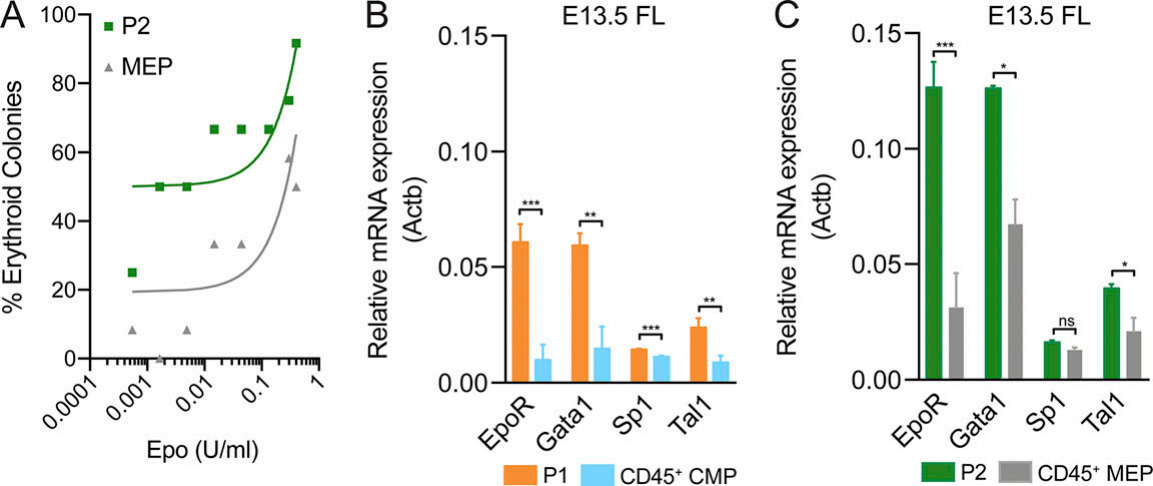
**P1/P2 cells have higher sensitivity to Epo than HSC derived progenitors.** **(A)** Frequency of erythroid colonies in P2 and MEP (CD45^+^) cells from E13.5 FL using serial dilutions of Epo. Representative plot of three independent experiments. Curves represent the linear regression of the data. **(B)** Quantitative RT-PCR analysis of the expression levels of *Epor* and its regulators *Gata1*, *Sp1*, and *Tal1* in P1 and CD45^+^ CMPs (*n* = 3). **(C)** Quantitative RT-PCR analysis of the expression levels of *Epor* and its regulators *Gata1*, *Sp1*, and *Tal1* in P2 and CD45^+^ MEPs (*n* = 3). In each experiment, cells were sorted from the same embryos. Statistical significance was assessed using paired *t* tests. *, P < 0.05; **, P < 0.01; ***, P < 0.001. Data are represented as mean ± SD.

FL stroma produces Epo, essential for erythrocyte production, albeit at lower concentrations than the adult kidney (Suzuki et al., 2011). Embryonic progenitors have been reported to react to lower concentrations of Epo than their adult counterparts (Rich and Kubanek, 1980). We compared erythrocyte production from P2 of YS origin with that from CD45^+^ MEPs of HSC origin from the same FL (Fig. 7 A). HSC-derived MEPs were about twofold less efficient in generating erythrocytes than YS-derived P2 at limiting levels of Epo and required >10-fold higher concentrations to reach 50% of erythrocyte colony formation. P2 cells exhibited higher sensitivity to Epo, and both P1 and P2 cells express higher levels of Epo receptor (EpoR) than CD45^+^ CMPs or MEPs of HSC origin from the same FL (Fig. 7, B and C). *Gata1*, *Sp1*, and *Tal1* transcripts that regulate *Epor* expression (Zon et al., 1991; Feng and Kan, 2005; Rogers et al., 2012) were increased in P1 and P2 when compared with CD45^+^ CMPs and CD45^+^ MEPs, respectively (Fig. 7, B and C). These results provide experimental evidence for the mechanism controlling the selection of YS-derived over HSC-derived progenitors in fetal erythropoiesis.

## Discussion

Here, we describe a population of CD45^−^Kit^+^ (P1/P2) hematopoietic cells unique to FL, found from E11.5 up until birth and that, at its peak (around E14.5), represents a major population comprising >70% of the CD45^−^Ter119^−^ FL cells and 10–15% of total FL cells. These cells give rise to erythroid colonies (50–70%) at higher frequencies than adult BM MEPs (∼15%; Akashi et al., 2000). The majority of CD45^−^Kit^+^ cells express CD24 and CD71, the transferrin receptor only found in erythroid progenitors (Dong et al., 2011).

Gene expression analysis and in vitro assays indicated a developmental progression where P1 cells further develop into P2 and later into P3 cells before acquisition of Ter119 expression. P3 cells did not generate colonies in vitro and expressed the erythroid transcripts at levels similar to Ter119^+^ cells, a stage at which they also express *Hbb-b1*, low levels *of Hbb-bh1*, and undetectable *Hbb-y* transcripts, indicating they are within the definitive erythroid lineage. Of note, P1 cells are among the most actively proliferative FL progenitors, indicating that they can considerably expand before terminal differentiation.

YS-derived primitive erythrocytes and megakaryocytes (Tober et al., 2008) and tissue-resident macrophages are *c-Myb* independent, and so is EMP generation (Schulz et al., 2012). Accordingly, CD45^+^ cells present in the FL of *c-Myb* mutants representing tissue resident macrophages were not affected (Schulz et al., 2012). Ter119^+^ cells, however, were drastically decreased in the FL of *c-Myb* mutants, and P1 cells were undetectable (Schulz et al., 2012). Ter119^+^ cells in the *c-Myb*^−/−^ FL expressed embryonic globins (εy and βh1), consistent with their primitive origin, and did not express *Klf1*, a transcription activator of the β-globin promoter essential for the transition from embryonic to adult hemoglobin expression (Perkins et al., 1995; Perkins et al., 2016). c-Myb induces proliferation of erythroid progenitors and c-Kit expression, and therefore HSC-independent erythroid cells are affected by *c-Myb* mutations (Vegiopoulos et al., 2006). Recently c-Myb was shown to control the expression of *Klf1* and *Lmo2* required for erythropoiesis, offering an explanation for the differential impact of c-Myb inactivation in the progeny of YS-derived EMPs (Bianchi et al., 2010).

Single injections of OH-TAM at E8.5 in Csf1r^MeriCreMer^Rosa26^YFP^ mark exclusively YS-derived cells and their progeny, among which is the microglia (Gomez Perdiguero et al., 2015). In these mice, P1 and P2 cells are marked at levels similar to the microglia 3 d after OH-TAM, indicating they are the progeny of YS EMPs. Consistent with the lineage relationship previously established, the frequency of labeled P1/P2 cells decreased with time after injection, whereas the frequency of labeled erythroblasts in FL and later in blood increased. EMPs emerge in the YS between E8.5 and E10.5, and a single injection of OH-TAM at E8.5 will reach the highest circulating levels of the drug 6 h later, rapidly decreasing thereafter (Zovein et al., 2008). Only a fraction of EMPs or already differentiated myeloid progeny that maintain *Csf1r* expression will be labeled with YFP. By contrast, differentiation into erythroid progenitors results in the loss of *Csf1r* expression, thus explaining the decreasing frequency of labeled immature erythroid progenitors with time. P1, P2, and P3 and erythroblasts are labeled at similar levels to those found in microglia up until E14.5, a time point after which labeling of erythroblasts and erythrocytes in blood increases. Similar results were obtained by analyzing Cdh5^CreERT2^Rosa26^YFP^ embryos, in which a pulse with OH-TAM at E7.5 labels the hemogenic endothelium, which gives rise to YS progenitors but does not generate HSCs (Gentek et al., 2018).

An opposite kinetic is found in Flt3^Cre^Rosa26^YFP^ mice, where Flt3-expressing cells and their progeny are permanently labeled with YFP. By E16.5, where equivalent frequencies of LSKs, CMPs, and GMPs are YFP^+^, only a small frequency of erythroid progenitors including MEPs and virtually undetectable frequencies of P3 or erythroblasts are labeled. By E18.5, MEPs were labeled at similar levels to those of CMPs, although the erythroblast compartment still shows a modest contribution of *Flt3*-expressing progenitors. Because lymphoid progenitors persistently express *Flt3* after commitment, they are the highest labeled population in this model and were excluded from the analysis. It has been recently described that MEPs can bypass the stage of *Flt3*-expressing MPPs and would therefore be undetected in this model (Carrelha et al., 2018). Cdh5^CreERT2^Rosa26^YFP^ embryos pulsed with OH-TAM at E10.5 where HSC-derived hematopoiesis is labeled (Zovein et al., 2008) exhibited a minor fraction of YFP^+^ erythroblasts or erythrocytes at any time point. By contrast, nonerythroid CD45^+^Kit^−^ mature populations in FL do not appear to be preferentially originated from one of the sources. We thus observed consistent results in both models and excluded the possibility of underestimating the contribution of HSCs to fetal erythrocytes due to the bypass of Flt3^+^ progenitors by MEPs.

**Figure 8.**
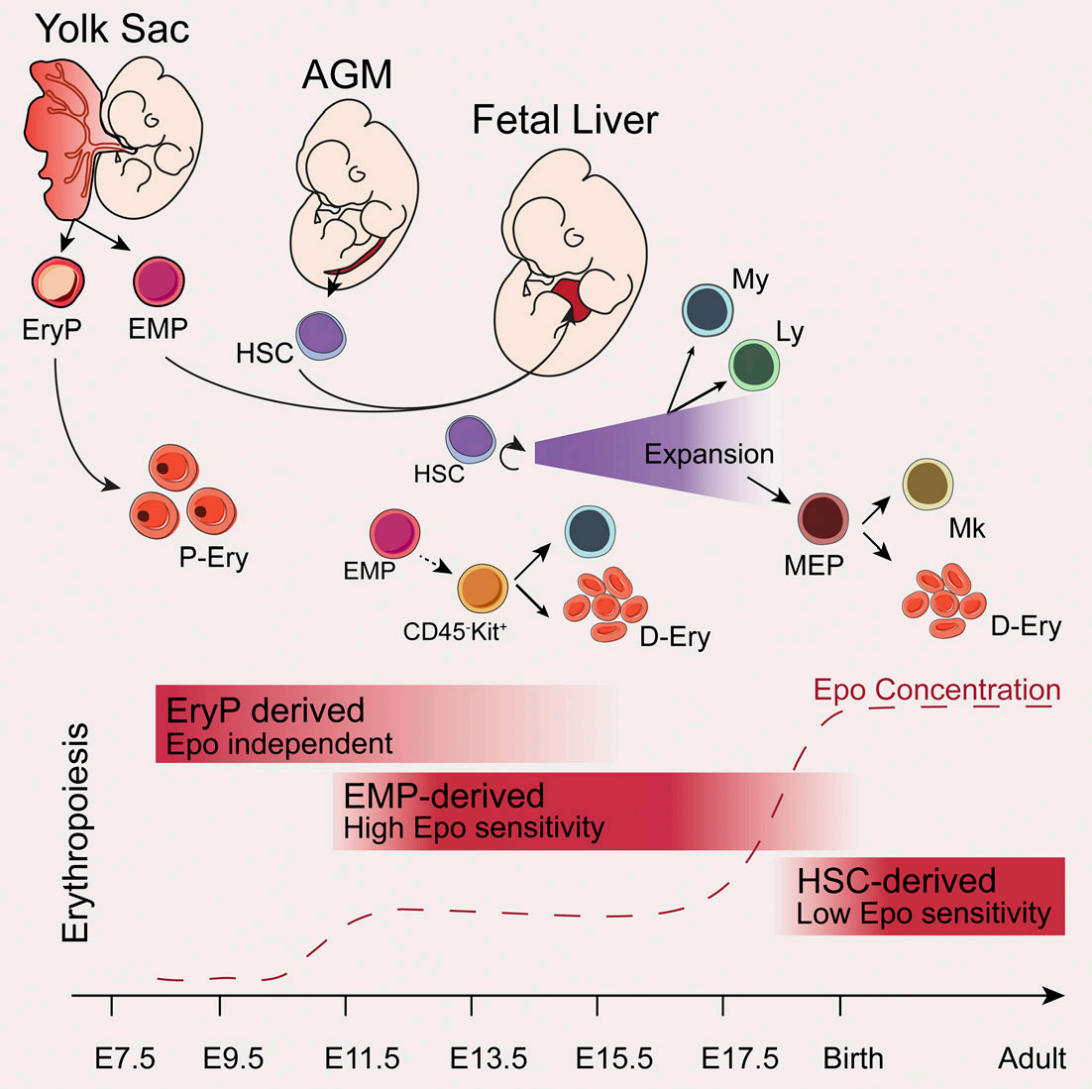
**Model of embryonic erythropoiesis. **Primitive erythroid progenitors (EryP) are first generated in the YS (E7.5) and give rise to primitive nucleated erythrocytes (P-Ery), still found in low frequencies at birth. A second wave of progenitors emerges in the YS (E8.5) as EMPs. EMPs migrate to the FL, where they differentiate into highly proliferative erythroid (CD45^−^Kit^+^) progenitors that sustain erythropoiesis during embryonic life. HSCs generated in the aorta-gonads-mesonephros (AGM; E9.5-E11.5) migrate to the FL, where they expand and differentiate into myeloid and lymphoid lineages. Contribution of HSCs to the erythroid lineage is only detected after birth. YS-derived progenitors respond to lower levels of Epo than their HSC counterparts and have a selective advantage in FL, where Epo levels are lower than in adult BM. D-Ery, definitive erythrocytes; Ery, erythroid cell; Ly, lymphoid lineage; Mk, megakaryocyte; My, myeloid lineage.

HSCs in FL expand but also differentiate, giving rise to multilineage progeny that comprise CMPs, GMPs, and lymphoid progenitors. However, our data show that despite a rapid differentiation of FL HSCs, they do not contribute significantly to the erythroid compartment before birth, and therefore in vivo embryonic HSC differentiation is skewed (Fig. 8). The FL stromal microenvironment can sustain erythropoiesis, and FL HSCs can differentiate into erythrocytes in vitro. We show that the low levels of Epo available in FL, before the kidney is competent to produce adult levels of this hormone, modulate the differentiation pattern of HSCs that do not produce MEPs and do not contribute to erythropoiesis (Zanjani et al., 1981). Consistently, low levels of the EpoR regulator Gata1 result in embryonic anemia but normal adult erythropoiesis (McDevitt et al., 1997). Our observation that YS-derived erythrocyte progenitors express higher levels of *Gata1*, *Tal1*, and consequently *Epor* is also consistent with an advantage of embryonic vs. adult erythroid progenitors. Large numbers of expanding YS-derived erythrocyte progenitors efficiently outcompete HSC progeny in an environment where resources for erythroid differentiation are limiting.

These results reinforce the notion that in contrast to what has been accepted, YS hematopoiesis is not only devoted to providing oxygen to the embryo before HSCs differentiate in FL but also to sustaining embryonic survival until birth (Fig. 8). FL was shown to be a less efficient environment for cytokine production than BM. Recently, low levels of interleukin-7 in FL were identified as the selective mechanism that resulted in autoreactive properties of B1 B cells (Wong et al., 2019). It is therefore tempting to speculate that low cytokine production is a general characteristic of FL cells with important implications for fetal hematopoiesis. In humans, kidney-derived Epo is also only produced after birth, offering an explanation for why premature neonates develop severe anemia (Widness, 2008).

In this line, a recent report analyzing human FL hematopoiesis indicates that all cells in the erythrocyte lineage, similar to the observation in the mouse reported here, do not express CD45 at stages ranging from 7–17 wk after conception (Popescu et al., 2019).

Our observations may also help clarify why patients with mutations in *Epo* that alter the kinetics of receptor binding only show postnatal anemia (Kim et al., 2017). These observations suggest that fetal erythropoiesis originates in the YS also in humans and will impact our understanding of embryonic hematopoiesis in general and in the pathogenesis of infant erythrocyte abnormalities.

## Materials and methods

### Mice

C57BL/6J mice were purchased from Envigo. Ubiquitin-GFP (Schaefer et al., 2001) mice used for transplantation experiments were a kind gift from P. Bousso (Institut Pasteur, Paris, France). Myb^−/−^, Csf1r^MeriCreMer^, Flt3^Cre^, Rosa26^YFP^, and Cdh5^CreERT2^ mice have been previously described (Gomez Perdiguero et al., 2015; Gentek et al., 2018). 6–8-wk-old mice or timed pregnant females were used. Timed pregnancies were generated after overnight mating. The following morning, females with vaginal plug were considered to be at E0.5. Recombination in Csf1r^MeriCreMer^Rosa26^YFP^ was induced by a single injection at E8.5 of 75 µg/g body weight of OH-TAM (Sigma-Aldrich; Cat# H7904), supplemented with 37.5 µg/g body weight progesterone (Sigma-Aldrich; Cat# P3972), as described (Gomez Perdiguero et al., 2015). Recombination in Cdh5^CreERT2^ was induced by a single injection at E7.5 or E10.5 of 1.2 mg of OH-TAM, supplemented with 0.6 mg progesterone, as described (Gentek et al., 2018).

All animal manipulations were performed under specific pathogen–free conditions and were performed according to the ethics charter approved by French Agriculture Ministry and to the European Parliament Directive 2010/63/EU.

### Cell suspension

E11.5–E18.5 FLs were dissected under a binocular magnifying lens. FLs were recovered in HBSS (GIBCO BRL; Cat# 24020–091) supplemented with 1% FCS (Eurobio; Cat# CVFSVF000U) and passed through a 26-gauge needle of a 1-ml syringe to obtain single-cell suspensions. Before staining, cell suspensions were filtered with a 100-µm cell strainer.

### Flow cytometry and cell sorting

For sorting, FLs were depleted (Ter119^+^CD45^+^) using MACS Columns (Miltenyi Biotec; Cat# 130–042-401). Cell suspensions were stained for 20–30 min at 4°C in the dark with the following antibodies when appropriate: CD11b (M1/70), CD16/32 (93 or 2.4G2), CD19 (1D3), CD24 (M1/69), CD31 (MEC13.3 or 390), CD324 (DECMA-1), CD34 (RAM34), CD41 (MWReg30), CD45 (30-F11 or 104), CD54 (YN1/1.7.4), CD71 (C2), Gr1 (RB6-8C5), Il7ra (A7R34), Ki-67 (SolA15), c-Kit (2B8), Sca1 (D7), and Ter119 (obtained from SONY, BD Biosciences, Biolegend, or eBioscience). Biotinylated antibodies were detected by incubation for 15 min at 4°C in the dark with streptavidin. Antibodies to lineage markers included anti-Ter119 (TER-119), anti-Gr1 (RB6-8C5), anti-CD19 (6D5), anti-CD3 (145-2C11), anti-CD4 (GK1.5), anti-CD8 (53–6.7), anti-NK1.1 (PK136), anti-Il7r (A7R34), anti-TCRβ (H57-597), anti-TCRγδ (GL3), and anti-CD11c (N418) obtained from SONY, BD Biosciences, Biolegend, or eBioscience. Stained cells were analyzed on a custom BD LSR Fortessa or BD FACSymphony and were sorted with a BD FACSAria III (BD Biosciences) according to the guidelines for the use of flow cytometry and cell sorting (Cossarizza et al., 2019). Data were analyzed with FlowJo software (BD Biosciences; v.10.5.3) or R packages as described in Bioinformatic analysis.

### RNA-seq and analysis

Total RNA from sorted E14.5 FL cells was extracted using the RNeasy Micro kit (Qiagen) following the manufacturer’s instructions, and ribosomal RNA sequences were eliminated by enzymatic treatment (Zap R, Clontech). cDNA libraries were generated using the SMARTer Stranded Total RNA-Seq Kit–Pico Input Mammalian (Clontech). The single-read RNA-seq reads were aligned to the mouse reference genome GRCm38 using STAR. The numbers of reads aligned to genes were counted using FeatureCounts (Liao et al., 2014). The R package DESeq2 (Love et al., 2014) was used to normalize reads and identify differentially expressed genes with statistical significance using the negative binomial test (P < 0.05; Benjamini–Hochberg correction).

Enrichr was used to perform gene set enrichment analysis of the highly differentially expressed genes in P2 vs. CD324^+^ cells (greater than twofold differential expression; the gene list is available in Table S1; Chen et al., 2013). The top 10 terms from the Gene Ontology Biological Process 2018 and ARCHS4 Tissues were retrieved. Expression datasets are available in GEO under accession no. GSE138960.

### Gene expression by RT-PCR

Cells were sorted directly into lysis buffer, and mRNA was extracted (RNeasy Micro Kit (Qiagen; Cat# 74004), reverse-transcribed (PrimeScript RT Reagent Kit (Takara Bio; Cat# RR037A), and quantitative PCR (qPCR) was performed with Power SYBR Green PCR Master Mix (Applied Biosystems; Cat# 4304437). Primers used in this study were as follows: *Actb*: 5′-GCT​TCT​TTG​CAG​CTC​CTT​CGT-3′ and 5′-ATC​GTC​ATC​CAT​GGC​GAA​CT-3′; *Bmi1*: 5′-ATC​CCC​ACT​TAA​TGT​GTG​TCC​T-3′ and 5′-CTT​GCT​GGT​CTC​CAA​GTA​ACG-3′; *Epor*: 5′-GGG​CTC​CGA​AGA​ACT​TCT​GTG-3′ and 5′-ATG​ACT​TTC​GTG​ACT​CAC​CCT-3′; *Gata1*: 5′-ATC​AGC​ACT​GGC​CTA​CTA​CAG​AG-3′ and 5′-GAG​AGA​AGA​AAG​GAC​TGG​GAA​AG-3′; *Hbb-b1*: 5′-GCA​CCT​GAC​TGA​TGC​TGA​GAA-3′ and 5′-TTC​ATC​GGC​GTT​CAC​CTT​TCC-3′; *Hbb-bh1*: 5′-TGG​ACA​ACC​TCA​AGG​AGA​CC-3′ and 5′-TGC​CAG​TGT​ACT​GGA​ATG​GA-3′; *Hbb-y*: 5′-TGG​CCT​GTG​GAG​TAA​GGT​CAA-3′ and 5′-GAA​GCA​GAG​GAC​AAG​TTC​CCA-3′; *Klf1*: 5′-AGA​CTG​TCT​TAC​CCT​CCA​TCA​G-3′ and 5′-GGT​CCT​CCG​ATT​TCA​GAC​TCA​C-3′; *Lmo2*: 5′-ATG​TCC​TCG​GCC​ATC​GAA​AG-3′ and 5′-CGG​TCC​CCT​ATG​TTC​TGC​TG-3′; *c-Myb*: 5′-AGA​CCC​CGA​CAC​AGC​ATC​TA-3′ and 5′-CAG​CAG​CCC​ATC​GTA​GTC​AT-3′; and *Runx3*: 5′-CAG​GTT​CAA​CGA​CCT​TCG​ATT-3′ and 5′-GTG​GTA​GGT​AGC​CAC​TTG​GG-3′. qPCR reactions were performed on a Quantstudio3 thermocycler (Applied Biosystems). Gene expression was normalized to that of β-actin, and relative expression was calculated using the 2^−ΔCt^ method.

### Imaging flow cytometry analysis

E13.5 FL cells were stained with the surface markers CD45 BV605 (104; 1:50 dilution) and Ter119 biotin (TER-119; 1:100 dilution) followed by incubation with streptavidin PE-Cy7 (1:100 dilution), CD71 PE (C2; 1:100 dilution), CD24 BV510 (M1/69; 1:50 dilution), Kit Pacific Blue (2B8; 1:20 dilution), and the RNA Dye Thiazole Orange (Sigma-Aldrich; Cat# 390062). Prior to acquisition, nuclei were stained with 20 µM DRAQ5 (Biostatus; Cat# DR0200) and filtered with 100-µm mesh. Data acquisition was performed using an ImageStream^x^ Mark II Imaging Flow Cytometer (Amnis, Luminex Corp.) using 405-nm, 488-nm, 561-nm, and 642-nm excitation lasers and the 40× magnification collection optic. Laser powers were set in order to maximize signal resolution but minimize any saturation of the charge-coupled device camera with brightfield images collected in channels 1 and 9. A minimum of 100,000 cell events was collected per sample. To calculate spectral compensation, single-stained cells were acquired with the brightfield illumination turned off. Spectral compensation and data analysis were performed using the IDEAS analysis software (Luminex Corp.; v.6.2.64).

### EdU incorporation and cell cycle analysis

EdU detection was done using the Click-iT EdU Pacific blue flow cytometry assay kit (Invitrogen; Cat# C10418). The cell cycle was analyzed after fixation with the Fixation/Permeabilization kit (Invitrogen; Cat#72-5775-40) and staining with Ki67 (SolA15). DAPI was added 7 min before analysis.

### In vitro liquid and semi-solid cultures

For limiting dilution analysis, sorted cells were plated in 1:3 diluting densities starting at 27 cells/well until 1 cell/well was reached in complete medium OPTI-MEM with 10% FCS, penicillin (50 U/ml), streptomycin (50 µg/ml), and β-mercaptoethanol (50 µM) supplemented with a saturating amount of the following cytokines: macrophage CSF, GM-CSF, c-Kit ligand, Epo (R&D Systems; 959-ME), and thrombopoietin (R&D Systems; 488-TO) for myeloid and erythroid differentiation. Except if stated otherwise, cytokines were obtained from the supernatant of myeloma cell lines (provided by F. Melchers, Deutsches Rheuma-Forschungszentrum, Berlin, Germany) transfected with cDNA encoding those cytokines. After 5–7 d, wells were assessed for the presence of hematopoietic colonies. Cell frequencies, determined with extreme limiting-dilution analysis software from the Walter and Eliza Hall Institute Bioinformatics Division, are presented as the number of positive wells and the number of total tested wells (Hu and Smyth, 2009).

For colony-forming assays, sorted cells were plated at 100 cells/35 mm culture dishes in duplicate in Methocult M3434 (StemCell Technologies; Cat# 03434) as described by the manufacturer. CFU-Es were assessed at 3 d and remaining colonies at 7 d.

### In vivo analysis of lineage potential

Pregnant WT females were anesthetized by intraperitoneal injection of a solution of ketamine 10 mg/ml plus xylazine 1 mg/ml diluted in PBS (50–100 µl per 10 g body weight), using a 1-ml syringe. E13.5 GFP^+^ Kit^+^CD24^+^ or CD45^+^LK FL cells from UBC-GFP embryos were purified and injected intraperitoneally into recipient E13.5 WT embryos (20,000 cells/embryo) of anesthetized females. Sham controls were performed. The FL and the fetal blood of injected and control embryos were analyzed at E16.5.

### Multiplex single-cell qPCR

Single cells were sorted directly into 96-well plates loaded with RT-STA Reaction mix (CellsDirect One-Step qRTPCR Kit; Invitrogen; Cat# 11753–500; according to the manufacturer’s procedures) and 0.2× specific TaqMan Assay mix and were kept at −80°C at least overnight. TaqMan probes used were as follows: *Bmi1*: Mm00776122_gH; *Cebpa*: Mm00514283_s1; *Csf1r*: Mm01266652_m1; *Csf2ra*: Mm00438331_g1; *Csf3r*: Mm00432735_m1; *Epor*: Mm00833882_m1; *Gata1*: Mm01352636_m1; *Gata2*: Mm00492301_m1; *Hbb-b1*: Mm01611268_g1; *Hbb-bh1*: Mm00433932_g1; *Hbb-y*: Mm00433936_g1; *mKi-67*: Mm01278617_m1; *Kit*: Mm00445212_m1; *Klf1*: Mm00516096_m1; *Lmo2*: Mm01281680_m1; *Ly6c*: Mm03009946_m1; *Mpl*: Mm00440310_m1; *Myb*: Mm00501741_m1; *Runx1*: Mm01213404_m1; *Runx2*: Mm03003491_m1; *Runx3*: Mm00490666_m1; *Tal1*: Mm01187033_m1; *Zfpm1*: Mm00494336_m1; *Actb*: Mm01205647_g1; *Gapdh*: Mm99999915_g1; and *Hprt*: Mm03024075_m1. For each subset analyzed, a control well with 20 cells was also sorted. Pre-amplified cDNA (20 cycles) was obtained according to manufacturer’s instructions and was diluted 1:5 in Tris-EDTA (TE) buffer for qPCR. Multiplex qPCR was performed using the microfluidics Biomark HD system for 40 cycles (Fluidigm) as previously described (Chea et al., 2016). The same TaqMan probes were used for both RT/preamp and qPCR. Only single cells for which at least two housekeeping genes could be detected before 20 cycles were included in the analysis.

### Bioinformatic analysis

Flow cytometry data analysis was performed in FCS files of live CD45^−^Ter119^−^ cell fractions using R packages “Rtsne,” “Rphenograph,” and “pheatmap” using Rv3.5.0. Gene expression raw data (BioMark; Fluidigm) of single cells was normalized with Gapdh and β-actin. Heatmaps and hierarchical clustering were generated using R packages “pheatmap” and “Rphenograph” (Levine et al., 2015).

### Quantification and statistical analysis

All results are shown as mean ± SD. Statistical significance was determined using one-way ANOVA followed by Tukey’s multiple comparison test where a P value of <0.05 was considered significant and a P value >0.05 was considered not significant.

### Online supplemental material

Fig. S1 shows the phenotype of E12.5, E14.5, and E18.5 FL and adult BM populations. Fig. S2 shows the gating strategies used to determine YFP-labeled hematopoietic populations in the Csf1r^MeriCreMer^Rosa26^YFP^ lineage tracing model with E8.5 OH-TAM injection. Fig. S3 shows the gating strategies used to determine YFP-labeled hematopoietic populations in the Cdh5^CreERT2^Rosa26^YFP^ fate-mapping model with E7.5 OH-TAM injection. Fig. S4 shows the gating strategies used to determine YFP-labeled hematopoietic populations in the Flt3^Cre^Rosa26^YFP^ fate-mapping model. Fig. S5 shows the gating strategies used to determine YFP-labeled hematopoietic populations in the Cdh5^CreERT2^Rosa26^YFP^ fate-mapping model with E10.5 OH-TAM injection. It also shows that YFP^−^ and YFP^+^ CMPs from Flt3^Cre^Rosa26^YFP^ E14.5 FL yielded similar frequencies of erythroid and myeloid cells and generated similar numbers of hematopoietic colonies. Table S1 shows the genes used for gene set enrichment analysis.

## Supplementary Material

Table S1 lists the 122 genes submitted to Enrichr, related to Fig. 1.
