# Supplementary material for: Yolk sac, but not hematopoietic stem cell–derived progenitors, sustain erythropoiesis throughout murine embryonic life
Source: J Exp Med. 2021 Feb 10;218(4):e20201729. doi: 10.1084/jem.20201729 (PMC7879581; doi:10.1084/jem.20201729)
Supplement: Table S1 — lists the 122 genes submitted to Enrichr, related to Fig. 1. [file JEM_20201729_TableS1.docx]

Table S1. List of the 122 genes submitted to Enrichr, related to Fig. 1

| A730089K16Rik | Cldn13 | Hk1 | Myb | Ripk3 | Tifa |
| --- | --- | --- | --- | --- | --- |
| Acsl6 | Cnr2 | Hsph1 | Myc | Rnf17 | Tmc8 |
| *Add2* | *Cox6b2* | *Ikzf1* | *Myh7b* | *Rpia* | *Treml2* |
| *Ampd3* | *Ctse* | *Il1rl1* | *Mylk3* | *Runx3* | *Trim58* |
| *Ank1* | *Def6* | *Inpp5d* | *Nefh* | *Sacs* | *Trpv2* |
| *Apbb1ip* | *Dyrk3* | *Itga4* | *Nxpe4* | *Samd14* | *Tspan32* |
| *Arap3* | *Epdr1* | *Kcnab2* | *Orc2* | *Samsn1* | *Tspo2* |
| *Arhgap15* | *Epor* | *Kcng2* | *Pcyt1b* | *Selplg* | *Ubash3a* |
| *Arhgap9* | *Ermap* | *Kcnn4* | *Pgm1* | *Slc14a1* | *Was* |
| *Arhgdig* | *Fam132a* | *Kit* | *Plxdc1* | *Slc29a1* | *Ydjc* |
| *Atp1b2* | *Fam78a* | *Klf1* | *Pmm1* | *Slc2a3* | *Zfp239* |
| *Bcap29* | *Fcho1* | *Lgals1* | *Prkar2b* | *Slc38a1* | *Zfp979* |
| *Bcl11a* | *Fermt3* | *Lmo2* | *Prps1* | *Slc38a5* |  |
| *Btk* | *Gata1* | *M1ap* | *Prss50* | *Slc7a1* |  |
| *C2cd4a* | *Gcnt1* | *Map4k1* | *Ptpn7* | *Slfn3* |  |
| *C530008M17Rik* | *Gdf3* | *Mc2r* | *Ptprcap* | *Sowaha* |  |
| *Car1* | *Gfi1b* | *Me2* | *Rasal3* | *Spire1* |  |
| *Casp3* | *Gm11427* | *Meiob* | *Rbm43* | *Spn* |  |
| *Cd37* | *Gm13212* | *Mfng* | *Recql4* | *Sppl2b* |  |
| *Ces2g* | *Gm15559* | *Mfsd2b* | *Rgs10* | *Tal1* |  |
| *Chst11* | *Gna15* | *Mns1* | *Rhag* | *Tarsl2* |  |
| *Cited4* | *Hesx1* | *Muc6* | *Rinl* | *Them6* |  |
